# Recent Development of Advanced Electrode Materials by Atomic Layer Deposition for Electrochemical Energy Storage

**DOI:** 10.1002/advs.201500405

**Published:** 2016-05-13

**Authors:** Cao Guan, John Wang

**Affiliations:** ^1^ Department of Materials Science and EngineeringNational University of SingaporeSingapore117574Singapore

**Keywords:** atomic layer deposition, electrochemical energy storage, electrode materials, nanostructures

## Abstract

Electrode materials play a decisive role in almost all electrochemical energy storage devices, determining their overall performance. Proper selection, design and fabrication of electrode materials have thus been regarded as one of the most critical steps in achieving high electrochemical energy storage performance. As an advanced nanotechnology for thin films and surfaces with conformal interfacial features and well controllable deposition thickness, atomic layer deposition (ALD) has been successfully developed for deposition and surface modification of electrode materials, where there are considerable issues of interfacial and surface chemistry at atomic and nanometer scale. In addition, ALD has shown great potential in construction of novel nanostructured active materials that otherwise can be hardly obtained by other processing techniques, such as those solution‐based processing and chemical vapor deposition (CVD) techniques. This review focuses on the recent development of ALD for the design and delivery of advanced electrode materials in electrochemical energy storage devices, where typical examples will be highlighted and analyzed, and the merits and challenges of ALD for applications in energy storage will also be discussed.

## 1 Introduction

This is an open access article under the terms of the Creative Commons Attribution License, which permits use, distribution and reproduction in any medium, provided the original work is properly cited.

With the rapid depletion of fossil fuels and ever‐increasing demand for clean and sustainable energy sources, development of advanced electrode materials for efficient energy storage has drawn much attention in recent years. Efficient electrochemical energy storage devices, including those of high energy density, power density and long device stability are desperately needed for electrical and hybrid vehicles, portable and wearable electronics, as well as large scale energy storage. Atomic layer deposition (ALD) is known to be a non‐solution nanotechnology for conformal deposition of nanoscale thin films and surface layers down to atomic layers with high uniformity and well‐controllable thickness and interface, and therefore gives rise to much improved device performance. It promises for applications in the next generation electrochemical energy storage.

This review focuses on the recent development of electrode engineering by ALD for electrochemical energy storage devices, where the unique principles and advantages of ALD technique will be discussed first. A detailed overview will then be presented on the key approaches for developing advanced electrodes by ALD for improved device performance. It will be presented in three parts: the first part will introduce ALD for surface modification; the second part will discuss active materials that have been successfully grown by ALD; they will be followed by a discussion on the novel nanostructures of active materials that can be uniquely delivered by ALD, such as those with 3D, core@shell and hollowed nanostructures, in the third part. Finally, in the conclusion and perspective, the success and challenges of ALD in advancing electrode materials will be summarized, together with a comprehensive outlook for future development.

### 1.1 Atomic Layer Deposition

**Figure 1:**
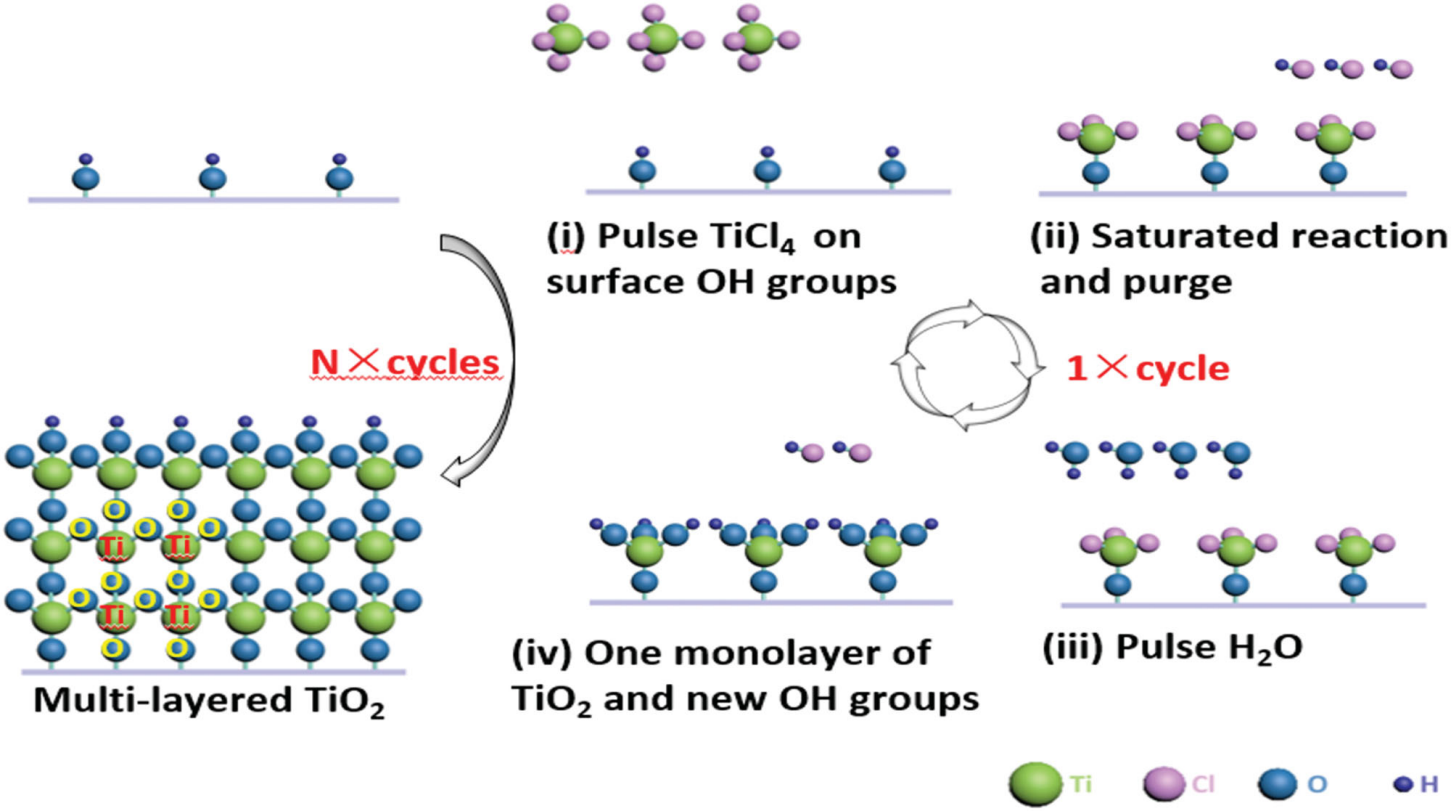
A schematic ALD process for depositing multi‐TiO_2_ layers using TiCl_4_ and water as the precursors on substrate with reactive OH* sites. A typical ALD cycles consists of: i) a pulse of TiCl_4_ precursor on substrate or pre‐deposited film reacts with the surface reactive sites (OH*) thus deposits Ti and introduce TiCl* reactive species; ii) upon the surface‐saturated reaction in (i), there is purge of the unreacted TiCl_4_ and by‐product HCl, leaving a new intermediate layer; iii) a pulse of H_2_O precursor reacts with TiCl* species to deposit O and supply OH* reactive sites; iv) the purge of oversupplied H_2_O and by product HCl, provides a clean surface with OH* species for the following cycles.

Atomic layer deposition (ALD), which is also historically named as atomic layer epitaxy (ALE), is a vapor‐based self‐terminating thin film growth technique, which can deliver a conformal coverage of layered materials with well‐controlled thickness, in particular on complex surfaces and 3D structures.^1^ It is an efficient and powerful deposition process that has been developed for deposition of various metals, metal oxides, metal nitrides, metal sulfides and compound materials. Since 1970s, ALD has steadily been established and commercialized for various thin films and surface coatings in chemical, mechanical and optical engineering as well as in microelectronics, where the best known examples are in electroluminescent displays and advanced high‐*k* metal oxides.^2^, ^3^ As ALD can effectively tailor the surface and porous structures of different materials, it has also been widely employed for the surface functionalization of materials for catalysis, fuel cells, batteries, and sensors, especially since 2000s.^4^ ALD process is based on successive cycles of self‐terminating gas‐solid surface reactions, where a typical cycle is composed of two or more pulses of precursors. Taking ALD TiO_2_ using two precursors (TiCl_4_ and H_2_O) as an example, the typical ALD process is schematically illustrated in **Figure** **1**, where the detailed procedure of one ALD cycle is as follows: (i) a pulse of TiCl_4_ precursor on substrate or pre‐deposited film reacts with the surface reactive sites (OH*), thus depositing Ti and introducing TiCl* reactive species; (ii) after the surface‐saturated reaction in (i), there is purge of the unreacted TiCl_4_ and by‐product HCl; (iii) a pulse of H_2_O precursor then reacts with TiCl* species to deposit O and supply OH* reactive sites; (iv) this is followed by purge of the oversupplied H_2_O and by‐product HCl, providing a clean surface with OH* species for the following cycles. The two half reactions in (i) and (iii) can thus be written as^3^, ^5^:

(1) $$\mathrm{TiOH*}+{\mathrm{TiCl}}_{4}\to {\mathrm{TiOTiCl}}_{3}^{*}+\mathrm{HCl}$$

(2) $$\mathrm{TiCl*}+H_{2}O\to \mathrm{TiOH*}+\mathrm{HCl}$$

The whole reaction process can be written as:

(3) $${\mathrm{TiCl}}_{4}+{\mathrm{2H}}_{2}O\to {\mathrm{TiO}}_{2}+\mathrm{4HCl}$$

**Figure 2:**
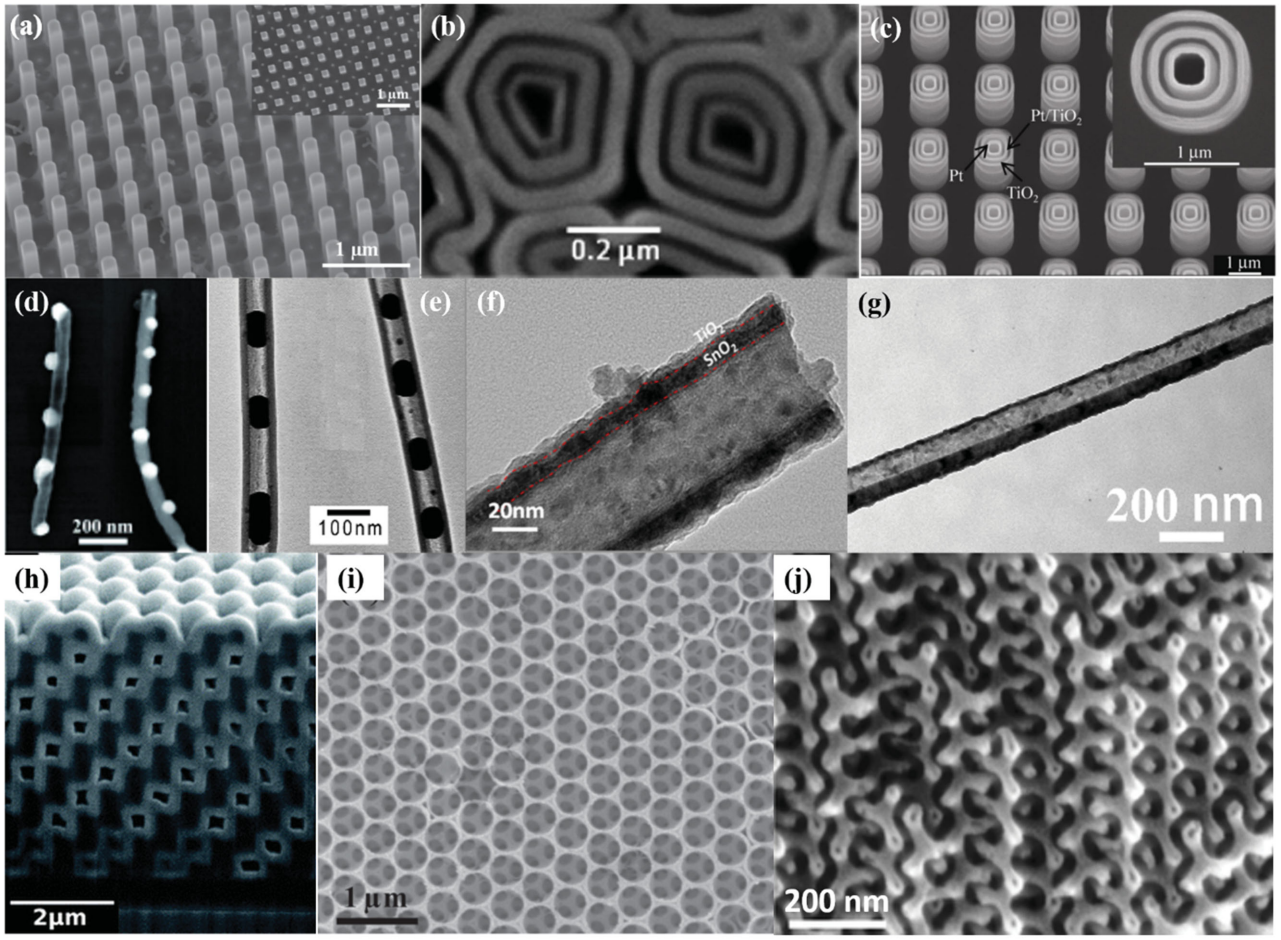
A collection of various nanostructures synthesised by ALD. a) SEM image of Pt nanotube arrays. Reproduced with permission.^122^ b) SEM image of triple coaxial HfO_2_ nanotubes separated by a gap and two sacrificial ALD Al_2_O_3_ layers. Reproduced with permission.^144^ Copyright 2010, American Chemical Society. c) SEM image of multi‐walled nested nanotube structures with ALD Pt and TiO_2_, where ALD Al_2_O_3_ was used as sacrificial spacer layers. Reproduced with permission.^145^ Copyright 2011, Springer. d) SEM images of one‐dimensional Au/Zn_2_SiO_4_ nanocomposites. Reproduced with permission.^133^ e) TEM image of Cu nanoparticles in ALD Al_2_O_3_ nanotube. Reproduced with permission.^146^ Copyright 2007, American Chemical Society. f) TEM image of ALD SnO_2_@TiO_2_ nanotube. Reproduced with permission.^127^ Copyright 2015, IOP Publishing. g) TEM image of ALD SnO_2_/TiO_2_ hollowed nanowire. Reproduced with permission.^131^ Copyright 2014, American Chemical Society. h) SEM image of ALD Al_2_O_3_ coated polymer template. Reproduced with permission.^147^ Copyright 2009, Royal Society of Chemistry. i) SEM image of ALD TiO_2_ inverse opals obtained from templates of PS spheres. Reproduced with permission.^148^ j) SEM image of ALD ZnO gyroid. Reproduced with permission.^149^ Copyright 2014, American Chemical Society.

Since the uniqueness of the ALD process, as compared with other gas‐phase deposition techniques, such as physical vapor deposition (PVD), chemical vapor deposition (CVD), and solution‐based deposition methods such as hydrothermal and sol–gel method, it demonstrates several apparent advantages, including well‐controllable thickness with high uniformity, excellent conformal deposition, and low temperature growth (normally below 300°C; some materials can be deposited at room temperature), even on complex surfaces and 3D substrates. **Figure** **2** shows a representative collection of electron microscopy images of various types of selected nanostructures, where ALD was involved in at least one of the fabrication steps. Compared with typical solution‐based deposition techniques, ALD relies on vapor‐phased surface reaction, thus the conformal deposition can be easily realised even on complex surfaces and 3D substrates. For example, conformal TiO_2_ inverse opals has been realized by ALD on templates of PS spheres (Figure 2i), and ZnO has been conformably coated on 3D polymer templates by ALD (Figure 2j). Since the amount of material deposition by ALD can be manipulated with the number of cycles, it delivers the desired high uniformity and accurate control in film thickness. For example, TiO_2_ and Pt nanotubes of considerable high uniformity have been formed with ALD Al_2_O_3_ sacrificial spacer layers (Figure 2c). As a typical example to show the structural uniformity, ALD Al_2_O_3_/ZnO film was demonstrated with a small surface roughness of ≈0.15 nm with the deposition thickness of ≈62 nm.^6^ Another advantage of ALD is its relatively low deposition temperature. Compared with conventional CVD techniques that are often conducted in the temperature range of 600–1050 °C,^7^ ALD can be run in temperatures below 300 °C. In addition, some materials can be effectively deposited below 100 °C or even at room temperature.^8^ This low temperature merit offers ALD a wide application potential, such as on temperature‐sensitive substrates, especially organic nanomaterials and biomaterials. For example, ALD ZnO was successfully operated at 70 °C on poly(methyl methacrylate) (PMMA) template,^9^ which helps to create a high‐density periodic ZnO nanopattern. In other reports, ALD Al_2_O_3_ and TiO_2_ were successfully deposited on biological macromolecules at 35 °C.^10^ Nanocrystalline In_2_O_3_ film with high conductivity could be deposited at a low temperatures of 100 °C.^11^ Last but not least, since ALD can offer conformal deposition on complex surfaces and 3D textures, it is of particular interest for use on the very wide range of different substrate types. With such apparent advantage, ALD has recently been investigated for materials in a wide spectrum of chemical, energy and environmental applications, such as catalysis, fuel cells, photovoltaics, batteries, supercapacitors, filtration devices, sensors and membranes. There were several review articles that introduced the fundamentals of ALD and its applications in nanotechnology,^4^, ^12^ and a few others on applications of ALD in lithium ion batteries and supercapacitors.^13^ Since the rapid development of ALD in energy applications, it is timely to review the recent progress, and therefore the present paper mainly focuses on the recent advances of ALD in design and fabrication of electrode materials for electrochemical energy storage devices.

### 1.2 Electrochemical Energy Storage

In the presently energy‐concerned society, potential energy crisis, globe warming and worsening environment have aroused huge attention to search for generation and storage of clean and sustainable energy at low cost.^14^ Among various energy storage techniques, electrochemical energy storage has been considered as one of the most promising, owing to the high efficiency, versatility, high mobility, low cost, and flexibility. There are different types of electrochemical energy storage devices that have been widely explored, for example, including rechargeable batteries and supercapacitors for electrical and hybrid vehicles, emergency power supply, portable electronics and wearable devices.^15^

In principle, electrochemical energy storage devices, such as rechargeable batteries and supercapacitors, keep energy in the format of electricity, which takes place through electrochemical processes by charge and discharge of electrons and ions, such as Li^+^, Na^+^, K^+^, H^+^ and OH^−^. A typical device of such electrochemical energy storage is composed of an anode, a cathode, and an electrolyte. During the discharge process (energy release), ions will desorb/extract from one electrode and transfer to the other, at the same time the electron thus generated flows pass through an external circuit; in the reverse charge (energy storage) process, the device will be under an external potential across the electrodes, thus the reverse electrochemical reactions (ions adsorption/insertion) takes place at the electrodes, and the electrochemical device becomes polarized with energy being stored after the charge process.

Rechargeable batteries can be further categorized as lead–acid, metal–air, nickel–iron, lithium–sulfur, sodium‐ion, and Li‐ion batteries (LIBs).^15^ Supercapacitors can be further classified into electric double layer capacitors (EDLCs) and pseudocapacitors. The electrochemical energy storage performance of both rechargeable batteries and supercapacitors is essentially determined by the electrode materials.^15^, ^16^ Even though there have been considerable investigation effects that are devoted to the design, selection and fabrication of advanced electrode materials, many challenges still exist for development of the next‐generation electrochemical energy storage devices.^14^, ^17^, ^18^ For example, as an anode in lithium ion batteries, carbonaceous materials can only provide limited capacities. Alloyed metal oxides and Si‐based materials suffer from huge volume changes, which result in poor cycling ability. Intercalation metal oxides usually show poor conductivity and low capacity. On the other hand, lithium ion battery cathode materials often suffer from limited rate performance and the side reaction with electrolyte. In Li–S batteries, the insulating sulfur and Li_2_S give rise to poor rate capability. The intermediate product of polysulfides can dissolve in electrolyte and damage the Li metal surface. In addition, the sulfur electrode undergoing huge volume expansion (≈76%) limits the cycling life. For application as supercapacitor electrode, carbonaceous materials have relatively low capacitance, while the metal oxides/hydroxides with low electric conductivity and low surface area usually show poor rate and cycling ability.

### 1.3 Main Advantages of ALD in Electrochemical Energy Storage

#### 1.3.1 Conformal Surface Protection for Enhanced Rate and/or Cycling Ability

Compared with other deposition techniques, such as the solution based sol–gel coating and hydrothermal reaction, the materials developed by ALD can offer much better conformal surfaces coating on electrodes and bring different surface chemical reactions, which serve as effective protection layers thus leading to better cycling ability. The ALD coating layers can be accurately controlled down to atomic level, which can benefit better rate capability. Detailed discussions on ALD surface protection of electrodes will be presented in Section 2.1, where selected examples will be given.

#### 1.3.2 Controllable Deposition for Optimised Power and Energy Density

The thickness/amount of active materials strongly influence the ion diffusion length and charge transfer path in electrochemical reactions, where in general a thicker layer of active material increases the energy density of the electrode but sacrifice the power density. Thus control in the amount of active material is essential in order to optimize the power and energy density. The thickness of active material by ALD can be easily manipulated by the number of cycles applied, which can therefore offer well‐tunable deposition of active materials with optimized overall performance. In addition, electrodes with a thin ALD layer offers the unique benefit for investigation and understanding of the charge transport and storage mechanism of the active materials. A detailed discussion on ALD of active materials for optimized electrochemical performance will be presented in Section 2.2.

#### 1.3.3 Uniform Layer for Novel Electrode Structure with Unique Properties

Since ALD gives rise to controllable uniform layers of active materials, it has been used to create novel nanostructures, some of which have been demonstrated with much better properties compared with those nanostructures obtained by other techniques, such as PVD and CVD. For example, the thin and uniform ALD layer can be ion‐exchanged leading to the formation of different active materials, while the structural uniformity is preserved. Examples of utilizing ALD in the development of unique electrode nanostructures will be further discussed in Section 2.3.

## 2 Utilizing ALD for Advanced Electrode Materials

As key components in almost all electrochemical energy storage devices, electrode materials are playing a determining role in the overall device performance. In this section, discussion will be focused on the development of ALD for advanced electrode materials. On the basis of the unique functions by ALD, the following three aspects will be presented: ALD for surface modification of electrodes; ALD for active materials; and ALD for construction of novel nanostructures. Typical examples with different ALD functions and the accordingly electrochemical performance are summarized in **Table** **1**, **Table** **2**, and **Table** 3.

**Table 1:** Summary of ALD for surface modification of electrodes

	Materials	ALD coating materials	Thickness/cycles	Application	Electrochemical performance	Ref.
1.	Nano‐LiCoO_2_	Al_2_O_3_	2 cycles	Li‐ion battery, cathode	a capacity of 133 mAh g^−1^ at 7.8 C; 100% capacity retention after 200 cycles.	22
2.	LiNi_0.5_Mn_1.5_O_4_ particles	Al_2_O_3_	4 cycles	Li‐ion battery, cathode	99.5% capacity retention after 80 cycles.	23
3.	LiNi_0.5_Mn_1.5_O_4_ particles	Al_2_O_3_	10 cycles	Li‐ion battery, cathode	91% capacity retention after 200 cycles 63% capacity retention after 900 cycles 90% capacity retention after 100 cycles at 55 °C.	24
4.	Li[Ni_1/3_Mn_1/3_Co_1/3_]O_2_	Al_2_O_3_	4 cycles	Li‐ion battery, cathode	92% capacity retention after 70 cycles at 55 °C (3.0–4.3 V, 1 C rate).	26
5.	0.3Li_2_MnO_3_0.7LiMn_0.60_Ni_0.25_Co_0.15_O_2_	Al_2_O_3_	10 cycles	Li‐ion battery, cathode	96.2 capacity retention after 25 cycles with 0.1 M HNO_3_ (%)	27
6.	Li_1.2_Ni_0.13_Mn_0.54_	Al_2_O_3_	2–3 nm	Li‐ion battery, cathode	82% capacity retention after 50 cycles.	28
	Co_0.13_O_2_					
7.	Li_1.2_Ni_0.13_Mn_0.54_Co_0.13_O_2_	TiO_2_	≈1 nm	Li‐ion battery, cathode	78% capacity retention after 50 cycles.	28
8.	LiMn_2_O_4_	Al_2_O_3_	6 cycles	Li‐ion battery, cathode	95.1% capacity retention after 50 cycles.	29
9.	LiNi_0.5_Mn_1.5_O_4_	LiAlO_2_	2 cycles	Li‐ion battery, cathode	92 mAh g^−1^ at C/3 rate, better than values from Al_2_O_3_ coating.	30
10.	LiMn_2_O_4_	Al_2_O_3_	10 cycles	Li‐ion battery, cathode	42.9 mAh g^−1^ at the 100th cycle, better than values from bare LiMn_2_O_4_ electrode	31
11.	LiNi_0.5_Co_0.2_Mn_0.3_O_2_	ZnO	8 cycles	Li‐ion battery, cathode	91.5% capacity retention after 60 cycles at 2 C; 92.5% capacity retention after 60 cycles at 55 °C with 5 C rate.	32
12.	LiMn_2_O_4_	ZrO_2_	6 cycles	Li‐ion battery, cathode	136.0 mAh g^−1^ at 1 C at 55 °C, 90.3 mAh g^−1^ after 100 cycles at 5 C at 55 °C.	33
13.	LiCoO_2_	TiO_2_ and Al_2_O_3_	10–500 cycles	Li‐ion battery, cathode	10 ALD cycles of Al_2_O_3_ and 50 ALD cycles of TiO_2_ coating results in best cycling ability.	34
14.	LiNi_0.5_Mn_1.5_O_4_	FePO_4_	10 cycles	Li‐ion battery, cathode	More than 80 mAh g^−1^ at 5 C; 91.96% capacity retention after 100 cycles at 0.5 C.	35
15.	LiCoO_2_	TiO_2_, ZrO_2_ and Al_2_O_3_	2 cycles	Li‐ion battery, cathode	Al_2_O_3_ coating‐best cycling ability (93.9% capacity retention after 100 cycles); ZrO_2_ coating‐the highest rate capability.	36
16.	LiMn_2_O_4_	CeO_2_	3 nm	Li‐ion battery, cathode	96% and 95% of capacity retention after	37
				1000 cycles with 1 C rate at room temperature and 55 °C.		
17.	Natural graphite	Al_2_O_3_	5 cycles	Li‐ion battery, anode	98% capacity retention after 200 cycles at 50 °C.	21
18.	Meso‐carbon microbeads	TiO_2_ and Al_2_O_3_	3 nm	Li‐ion battery, anode	Enhanced capacity (344 mAh g^−1^) compared to that of bare graphite (328 mAh g^−1^); 300 mAh g^−1^ after 40 cycles at 55 °C.	39
19.	Patterned silicon electrode	Al_2_O_3_	20 cycles	Li‐ion battery, anode	Initial charge capacity of 1125 mAh g^−1^, 1100 mAh g^−1^ after 100 cycles.	43
20.	SnO_2_ nanoparticles	Al_2_O_3_	2–20 cycles	Li‐ion battery, anode	2 ALD cycles on SnO_2_ particles with 4.1 nm average size shows the best cycling ability (883 mAh g^−1^ after 60 cycles).	44
21.	Amorphous Si thin film	Al_2_O_3_	2 cycles	Li‐ion battery, anode	Average capacity loss from cycle 2 to cycle 40 is about 0.28% per cycle; 2164 mAh g^−1^ at 100 C rate, 74% of its capacity at C/5.	45
22.	Fe_2_O_3_	Al_2_O_3_	2 cycles	Li‐ion battery, anode	100% capacity retention after 50 cycles at 0.5 mA g^−1^.	47
23.	Li_4_Ti_5_O_12_	ZrO_2_	1–50 cycles	Li‐ion battery, anode	Best initial capacity: 350 mAh g^−1^ for 2 cycles ALD coated electrode; Best rate capability: 106 mAh g^−1^ at 1600 mA g^−1^ for 5 cycles ALD coated electrode	49
24.	Activated carbons	Al_2_O_3_	2 nm	Supercapacitor	Excellent stability at 3V operation with 39% energy density enhancement from 2.5 V operation; 88% of capacitance was retained after 5000 cycles at 70 °C.	54
25.	Carbon nanotubes	V_2_O_5_	100 cycles	Supercapacitor	≈1550 F/g at 1 A g^−1^, 92% capacitance retention after 5000 cycles at 5 A g^−1^.	55
26.	Graphite foam/carbon nanotube	Fe_2_O_3_	100 cycles	Supercapacitor	≈470.5 mF cm^−2^, ≈4 times larger than that of bare carbon (≈93.8 mF cm^−2^); 111.2% capacitance retention after 50000 cycles.	56
27.	PANI nanofiber arrays	RuO_2_	100 cycles	Supercapacitor	710 F/g at 5 mV s^−1^; 88% capacitance retention after 10000 cycles.	59
28.	Sn nanoparticles	Al_2_O_3_	6.2–11.7 nm	Na‐ion battery	Initial charge capacity of 625 mAh g^−1^, and 650 mAh g^−1^ after 40 cycles.	63
29.	Na_2_C_8_H_4_O_4_	Al_2_O_3_	20 cycles	Na‐ion battery	52.8% of the capacity at 0.05 C maintained at 2 C; 79.8% capacity retention after 60 cycles.	62
30.	S‐infiltrated activated carbon fibers	Al_2_O_3_	30–50 cycles	Li–S battery	Capacity above 300 mAh g^−1^ maintained for 370–470 high‐temperature cycles.	70
31.	Mesoporous carbon black/S	Al_2_O_3_	2 cycles	Li–S battery	630 mAh g^−1^ after 70 cycles	73
32.	Reduced graphene oxide/S	ZnO	40 cycles	Li–S battery	998 mAh g^−1^ at a current density of 0.2 C; 846 mAh g^−1^ after 100 cycles.	72
33.	Porous carbon cloth/S	Al_2_O_3_	0.5 nm	Li–S battery	1136mAh g^−1^ at the 1st cycle, and 766mA h/g at the 40th cycle.	74
34.	Porous carbon	Pd	3 cycles	Li–O_2_ battery	6600 mAh g^−1^ at 100 mA g^−1^	77
35.	Carbon black	Pd + Al_2_O_3_	3 cycles+ 3 cycles	Li–O_2_ battery	a dramatic reduction in charge overpotential to ≈0.2V	78
36.	Mesoporous carbon	Pd + FeO*_x_*	1.8 nm	Li–O_2_ battery	Extends the cyclability from 16 to 68 cycles.	79
37.	Graphitized carbon black	Pd + ZnO	1 cycle + 2 cycles	Li–O_2_ battery	low charge potential of ≈ 2.8V	80

**Table 2:** Summary of ALD active materials for electrochemical energy storage

	ALD Active Materials	Substrate	Application	Electrochemical performance	Ref.
1.	Li_2_S	Meso carbon microbeans	Li–S battery	Stable capacity of ≈500 mAh g^−1^ over 500 cycles	95
2.	V_2_O_5_	Steel disc	Li‐ion battery	Maintain 20% of the initial 1 C capacity at 960 C; Maintain 80% of capacity at 120 C after 1500 cycles.	96
3.	TiO_2_	3D porous Au	Li‐ion battery	High power density of 13 KW Kg^−1^ with high energy density of 130 Wh Kg^−1^	97
4.	SnO_2_	Nano Ni foam	Li‐ion battery	Initial discharge capacity of 546 mAh g^−1^, and 505 mAh g^−1^ after 100 cycles at 500 mA g^−1^	98
5.	V_2_O_5_	AAO + ALD Ru	Li‐ion battery	50% of 1C capacity maintained at 150 C; 80% of initial capacity retained after 1 000 cycles at 5 to 25 C.	100
6.	LiFePO_4_	Carbon nanotube network	Li‐ion battery	≈150 mAh g^−1^ at 0.1 C and 71 mAh g^−1^ at 60 C; a high capacity retention of 120 mAh g^−1^ after 2000 cycles at 170 mA g^−1^.	104
7.	TiO_2_	Carbon cloth/carbon nanowire	Li‐ion battery	309 mAh g^−1^ at 0.2 C, 47 mAh g^−1^ at 50 C; 170 mAh g^−1^ after 8000 cycles at 10 C (only 0.0019% capacity decay per cycle).	105
8.	V_2_O_5_	Carbon sponge	Li‐ion battery	Areal capacity of 155 μAh cm^−2^ and a high power density of 21.7 mW cm^−2^.	107
9.	RuO*_x_*	Carbon nanotube	Supercapacitor	644 F/g with a high power density of 17 kW kg^−1^	109
10.	NiO	Nanoporous graphene	Supercapacitor	≈1897.1 F/g and cycling for 1500 cycles.	111

**Table 3:** Summary of utilizing ALD for the construction of advanced nanostructured electrodes

	ALD Materials	Functions of ALD	Application	Electrochemical performance	Ref.
1.	Pt	As a 3D current collector for electrochemical deposition of MnO_2_	Supercapacitor	810 F/g at 5 mV s^−1^; 68% capacitance retention from 2 to 100 A g^−1^; negligible capacitance loss after 8000 cycles.	122
2.	Ru	As a 3D current collector for ALD V_2_O_5_	Li‐ion battery	50% of 1C capacity maintained at 150 C; 80% of initial capacity retained after 1 000 cycles at 5 to 25 C.	100
3.	TiO_2_	As a 3D current collector for Fe_2_O_3_ and active materials	Li‐ion battery	530 mAh g^−1^ after 200 cycles at 200 mA g^−1^	123
4.	TiO_2_	As a 3D current collector for SnO_2_ and active materials	Li‐ion battery	530 mAh g^−1^ after 30 cycles	124
5.	SnO_2_/TiO_2_	As a 3D current collector and active materials	Li‐ion battery	778.8 mAh g^−1^ at 780 mA g^−1^	127
6.	Al_2_O_3_/TiO_2_	For construction of hollow nanostructures and surface protection	Supercapacitor	Specific capacitance increased from 518.9 F/g to 633.3 F g^−1^; 89.7% capacitance retention after 5000 cycles at 10 mA cm^−2^;	130
7.	ZnO/TiO_2_	For construction of hollow nanostructures and surface protection	Li‐ion battery	393.3 mAh g^−1^ is maintained after 1000 cycles.	131
8.	TiO_2_	For construction of hollow nanostructures, solid state diffusion reaction with Co(CO_3_)_0.5_(OH)_0.11_H_2_O	Li‐ion battery	≈600 mAh g^−1^ after 250 cycles	134
9.	SnO_2_	For construction of hollow nanostructures, form porous CoSnO_3_	Li‐ion battery	1162.1 mAh g^−1^ at 400 mA g^−1^; 59.9% capacity retention after 100 cycles.	135
10.	Al_2_O_3_	For construction of porous carbon nanoflakes	Supercapacitor	98.6% of capacitance maintained after 5000 cycles.	136
11.	ZnO	As a sacrificial layers for ion exchange to obtain Fe_2_O_3_	Li‐ion battery	785 mAh g^−1^ at 1C; 100% capacity retention after 500 cycles.	140
12.	ZnO/TiO_2_	As a sacrificial layers for ion exchange to obtain Fe_2_N and TiN	Supercapacitor	A high energy density of 15.4 Wh kg^−1^ and a high power density of 6.4 kW kg^−1^; 98% of capacitance maintained after 20000 cycles.	141

### 2.1 ALD for Surface Modification on Electrodes

The structure and surface chemistry of electrodes greatly influence the electrochemical performance in energy storage devices. This section will focus on ALD surface modification of different electrode materials, including lithium‐ion battery electrodes, supercapacitor electrodes, and electrodes for other energy storage devices, such as Na‐ion battery, Li–S and Li–O_2_ batteries. The unique function of ALD surface modification and the resultant contribution in enhancement of electrochemical performance will be highlighted.

#### 2.1.1 ALD Surface Modification for Lithium‐Ion Battery Cathodes

The direct contact of cathode materials with electrolytes can bring about side reactions that may cause slow degradation of electrodes, thus proper surface modification of the cathode materials have been conducted for improvement in electrochemical performance.^19^ The materials studied for surface modification include various metal oxides, phosphates and fluorides.^20^ These materials can be used as a physical protective layer or HF scavenger with improved ionic conductivity, thus much improved rate capability and cycling ability is achieved.^19^ Compared with traditional wet chemistry processes, such as sol–gel process for surface modification, ALD offers much uniform surface coverage on the electrode materials with well controlled thickness down to sub‐nanometers scales.

**Figure 3:**
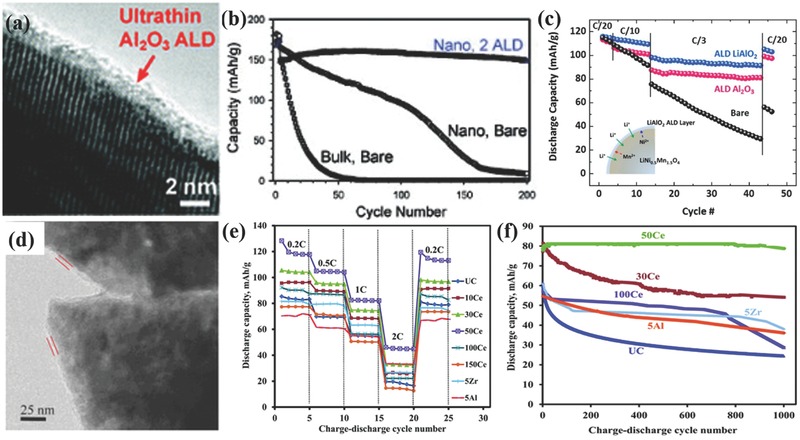
ALD surface modification for LIB cathodes and anodes. a) SEM image of ALD Al_2_O_3_ coated LiCoO_2_ nanoparticles. b) Cycling performance of bulk LiCoO_2_, uncoated nano‐LiCoO_2_, and Al_2_O_3_ coated nano‐LiCoO_2_. c) Room temperature cycling properties of LiNi_0.5_Mn_1.5_O_4_–graphite cells with different coating materials on electrodes: uncoated, cathode coated with LiAlO_2_ (cLiAlO_2_), anode coated with LiAlO_2_ (aLiAlO_2_), cathode coated with Al_2_O_3_ (cAl_2_O_3_), and anode coated with Al_2_O_3_ (aAl_2_O_3_). d) SEM image of 100 cycles of ALD CeO_2_ coated LiMn_2_O_4_ particles. e) Rate properties and f) cycling abilities of different electrodes: uncoated (UC), 5 cycles of ZrO_2_ (5Zr), 5 cycles of Al_2_O_3_ (5Al), and 10 (10Ce), 30 (30Ce), 50 (50Ce), 100 (100Ce), and 150 (150Ce) cycles of CeO_2_ coated LiMn_2_O_4_. a,b) Reproduced with permission.^22^ Copyright 2010, American Chemical Society. c)Reproduced with permission.^30^ Copyright 2014, American Chemical Society. d,e,f) Reproduced with permission.^37^

Jung et al. have demonstrated that direct ALD Al_2_O_3_ coating on micro‐powdered LiCoO_2_ electrode surfaces effectively protects the active material while maintaining an inter‐particle electronic pathway for high rate capability.^21^ It is believed that the ALD Al_2_O_3_ acts as an “artificial” solid electrolyte interphase (SEI), thus protects the inner active material from side reaction. However, if ALD Al_2_O_3_ was first deposited on LiCoO_2_ powder and then applied into an electrode, the electron conduction paths would be blocked thus the capacity would decrease rapidly. Scott et al. reported that the LIB performance of nano LiCoO_2_ powder‐based electrode could be significantly improved by ALD Al_2_O_3_ surface coating.^22^ As shown in **Figure** **3**a–b, 6 cycles of ALD Al_2_O_3_ with thickness of ≈1–2 nm gives rise to a uniform coverage on LiCoO_2_ powder particles. The ALD surface‐modified electrode maintained 100% capacity after 200 charge–discharge cycles at 2.8 C, in contrast to the capacity of bared LiCoO_2_ that had dropped to almost zero after the same number of cycles. The ALD surface coating also largely improves the rate capability of the LiCoO_2_ electrode. For example, two ALD cycles on Al_2_O_3_ coated electrode demonstrates a capacity of 133 mAh g^−1^ at 7.8 C, corresponding to about 250% improvement as compared to the bare LiCoO_2_ electrode. The uniform assembling of the thin ALD Al_2_O_3_ layer has effectively protected the electrode and maintained the high rate capability.

Other cathode materials, such as LiNi_0.5_Mn_1.5_O_4_,^23^, ^24^, ^25^ Li[Ni_1/3_ Mn_1/3_Co_1/3_]O_2_,^26^, ^27^ Li_1.2_Ni_0.13_Mn_0.54_Co_0.13_O_2_,^28^ LiMn_2_O_4_,^29^ have also shown much improvement with ALD Al_2_O_3_ surface modification. The ALD Al_2_O_3_ coating layer serves as an “artificial” SEI layer that suppresses the side reaction at high voltage thus improve the overall cathode performance.

Since the insulating nature of Al_2_O_3_ that retards Li^+^ diffusion process with low rate performance, other metal oxides, such as ALD LiAlO_2_, ZnO, ZrO_2,_ FePO_4_ and TiO_2_, have been investigated as surface modification on cathode materials.^30^, ^31^, ^32^, ^33^, ^34^, ^35^ For example, as shown in Figure 3c,^30^ Park et al. have investigated ALD LiAlO_2_ surface coating on LiNi_0.5_Mn_1.5_O_4_ electrode. Since the ionic conductivity of LiAlO_2_ is better than that of Al_2_O_3_, LiAlO_2_ surface‐modified electrode shows much improved rate capability and cycling ability when compared to Al_2_O_3_ coated electrodes. Using a commercial LiCoO_2_ electrode, Sun's group conducted a systematic investigation to compare the effects of ALD coating layers of TiO_2_, ZrO_2_ and Al_2_O_3_.^36^ Their results suggest that 2 ALD‐cycle coatings gives rise to the best improvement when compared with thicker coatings, and Al_2_O_3_ leads to the best cycling ability while ZrO_2_ shows the highest rate capability. Another interesting work from Liang's group reported an ALD CeO_2_ modified LiMn_2_O_4_ electrode, in which the pin‐hole free layer of CeO_2_ could not only protect the electrode from side reaction but also acted as an electronic path.^37^ Compared with Al_2_O_3_‐ and ZrO_2_‐ surface coated LiMn_2_O_4_, the electrode with CeO_2_ surface modification provides much improved rate capability at both room temperature and 55 °C. In addition, as shown in Figure 3d–f, CeO_2_ modified LiMn_2_O_4_ shows a stable cycling ability with a high capacity of 78 mAh g^−1^ being maintained even after 1000 cycles, which is also much improved compared with those of Al_2_O_3_ or ZrO_2_ ALD surface‐coated electrodes. The Li^+^ conductivity of ALD CeO_2_ ultrathin film effectively overcomes the tradeoff between Li^+^ diffusion and the “artificial” SEI protection.

#### 2.1.2 ALD Surface Modification For Lithium‐Ion Battery Anodes

As a practically valuable LIB anode material, graphite exhibits a theoretical capacity of 372 mAh g^−1^. However SEI layer can be formed on the graphite surface even during the first cycle, which brings potential safety issue, especially when the cell operation temperature is high.^38^ To protect graphite and reduce the SEI, surface modification works have been conducted with natural graphite to improve the cycling ability and high temperature safety. For example, Jung et al. have discovered that ALD Al_2_O_3_ on the surface of natural graphite could significantly improve its cycling ability and safety at high temperatures.^21^ It is demonstrated that the graphite electrode with 5 cycles of ALD Al_2_O_3_ maintains 98% of the initial capacity after 200 cycles of charge–discharge at 50 °C, while the capacity of the bare graphite electrode drops to only 26% under the same test condition. An interesting work from Wang's group observed the influence of ALD TiO_2_ surface coating on natural graphite,^39^ where they found that the ALD TiO_2_ served as artificial SEI to improve the stability of graphite electrode at the high temperature of 55 °C.

For LIB anodes, metal oxides have been considered as promising candidates for the next‐generation electrode materials, because of their generally large capacities and safety factor.^40^ Alloyed anode materials, such as Si and Sn‐based materials (such as SnO_2_ and Sn) have been investigated for lithium storage, due to their high capacities and relatively low onset potentials.^41^, ^42^ In the electrochemical process, however, these alloyed anodes undergo severe pulverization with huge volume expansion. When continuous SEI layers are formed, the cycling ability becomes very poor.^42^ Proper surface modification has thus been pursued to help solve the problem. As has been reported by He et al., an ALD Al_2_O_3_ coating layer on pattern Si column electrode not only prevents side reaction but also enhances the mechanic strength during the electrochemical reaction.^43^ The ALD Al_2_O_3_‐surface coated electrode also shows a higher Coulombic efficiency (remains above 99.5% after few cycles) than that of the uncoated one. ALD Al_2_O_3_ coated SnO_2_ has also been reported, where ALD Al_2_O_3_ can well serve as “artificial” SEI to protect side reaction from SnO_2_ and electrolyte.^44^ In addition, the cycling ability of the electrode is closely related to the size of SnO_2_ nanoparticles and the thickness of the protective layer. Since the volume expansion of SnO_2_ nanoparticles occurs during the cycling process, an protective ALD Al_2_O_3_ layer of appropriate thickness buffers the stress and strain thus to maintain the capacity. Further work on ALD surface coating on Si thin film^45^ and Si nanowires^46^ also demonstrates much improved cycling performance after the surface modification.

ALD surface coating helps improve on conversional and intercalation metal oxides, such as Fe_2_O_3_, MoO_3_ and Li_4_Ti_5_O_12_.^47^, ^48^, ^49^ As has been reported by Lipson et al., a relatively thick and rough SEI is formed on the surface of the uncoated MnO anode, while ALD Al_2_O_3_ coated MnO (with a thickness of 3 Å) can effectively prevent the formation of such SEI and maintain the capacity for more than 100 cycles.^50^ Sun's group have investigated the surface modification of ALD ZrO_2_ on intercalation Li_4_Ti_5_O_12_,^49^ in which the ultrathin ZrO_2_ layer is shown to prevent SEI for high stability and extend the voltage window of Li_4_Ti_5_O_12_ for high energy density.

There is no doubt that ALD surface modification on LIB electrodes can effectively suppress the side reactions, alleviate the stress and strain of electrodes, and prevent the decomposition of SEI especially at elevated temperatures. The surface reactions occurring in the anode and cathode are highly related,^51^ thus the LIB performance can be much improved by ALD surface coating on separator and both electrodes.^52^

#### 2.1.3 ALD Surface Modification for Supercapacitor Electrodes

Supercapacitors is another important class of widely investigated and employed electrochemical energy storage devices, which store charges either through absorption/desorption of ions by forming electric double layers (largely based on carbonaceous electrodes) or through fast reversible surface/near‐surface Faradic reactions (based on certain transition metal oxides/hydroxides and conducting polymers) or both.^16^ ALD surface coating technique on LIBs has been extended to supercapacitor electrodes, although the requirement for supercapacitors is quite different from that for LIBs. The performance of supercapacitors is largely relied on the surface/near surface reactions, thus in order to enhance the electrochemical performance, the coating materials should be thin and electrically conductive for fast surface reactions, and they preferably should be highly active and stable for electrochemical energy storage.

**Figure 4:**
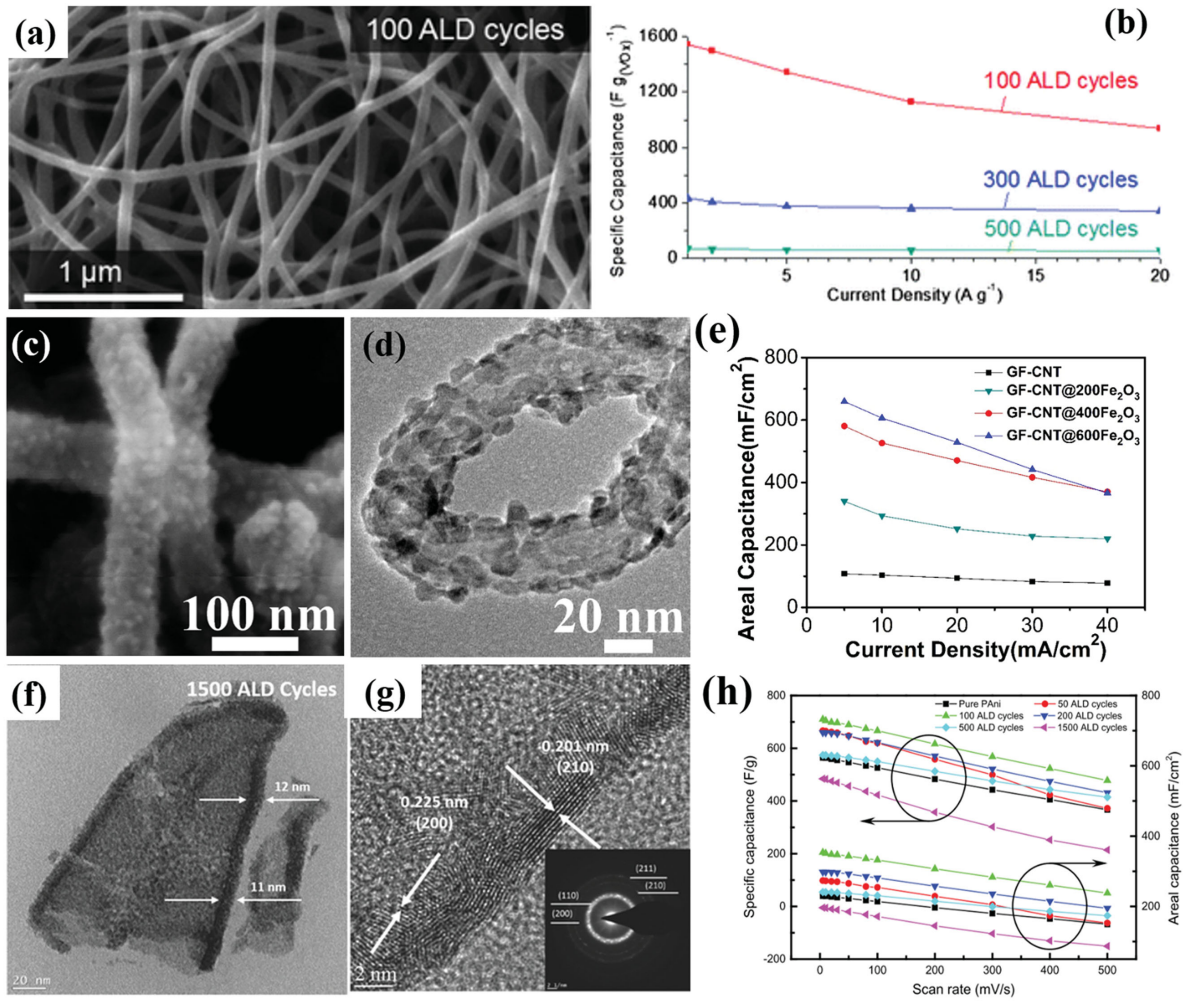
ALD surface modification of supercapacitor electrodes. a) SEM image of 100 cycle of ALD V_2_O_5_ coated CNTs. b) Specific capacitance of CNTs samples coated with 100, 300, and 500 cycles of ALD V_2_O_5_. c) SEM and d) TEM images of ALD Fe_2_O_3_ coated CNTs. e) Rate properties of graphite foam‐CNTs samples coated with 200, 400, and 600 cycles of ALD Fe_2_O_3_. f,g) HRTEM images with SAED pattern of PANI coated with ALD RuO_2_. h) Specific and areal capacitance as function of scan rate for PANI samples coated with different ALD cycles of RuO_2_. a,b)Reproduced with permission.^55^ Copyright 2012, Royal Society of Chemistry. c,d,e) Reproduced with permission.^56^ Copyright 2015, American Chemical Society. f,g,h) Reproduced with permission.^59^

Carbonaceous materials have been widely employed for supercapacitor electrodes, owing to several merits such as high electric conductivity, tunable high surface area, low cost, and high electrochemical stability.^53^ However, they exhibit relatively low capacitance and therefore low energy density. In order to enhance the energy density of carbon‐based materials, one approach is to expand their working voltage windows. However, carbon‐based materials are not stable at high‐voltages. To solve this problem, ALD Al_2_O_3_ layer has been investigated for coating on the active carbon surface, as has been reported by Hong et al.^54^ When tested in an organic electrolyte, the ALD coated electrode showed excellent stability with a 3 V voltage window, in which the energy density was 39% higher than that for 2.5 V voltage window. The ALD surface coating protects the surface functional groups and prevents the degradation of electrolyte. Another approach to enhance the energy density of carbonaceous materials is to deposit thin layers of anode materials (such as V_2_O_5_, Fe_2_O_3_, Bi_2_O_3_) on carbon surface, where the active material contributes additional capacitance for high energy density, but does not sacrifice much of the power density and cycling ability. For example, Boukhalfa et al. have studied ALD V_2_O_5_ coating on CNT electrodes, one sample of which is shown in **Figure** **4**a,b.^55^ The rather uniformly surface‐coated V_2_O_5_ provides more Faradic reaction for higher capacitance, and the CNTs ensure good electric conductivity for fast charge transfer. CNTs coated with 100‐ALD‐cycle V_2_O_5_ delivers a specific capacitance of 1400 F g^−1^ at 5 mV s^−1^, which is significantly improved when compared with that of the bare CNT electrode (≈30 F g^−1^). The V_2_O_5_ surface‐coated CNTs show a capacitance of above 360 F g^−1^, even when the current density is increased to 20 A g^−1^, demonstrating its high rate capability. On the other hand, too thick a V_2_O_5_ coating layer will block the active reaction sites and electron transport paths, and therefore leading to degraded electrochemical performance. Thus an optimized ALD thickness of V_2_O_5_ coating layer is needed. Wang's group has developed an anode material of ALD Fe_2_O_3_ deposited on hierarchical carbon support, one example of which is shown in Figure 4c–e.^56^ Different from the above mentioned ALD V_2_O_5_ which exhibited a 2D growth mode, ALD Fe_2_O_3_ was grown on CNTs under an island growth mode, where small nanoparticles of Fe_2_O_3_ were assembled rather uniformly on CNTs. When tested as the anode material in supercapacitors, the C@Fe_2_O_3_ delivered much improved capacitance than that of pure carbon substrate. For example, at the same current density of 20 mA cm^−2^ mA cm^−2^, the carbon electrode coated with 400‐ALD‐cycle of Fe_2_O_3_ showed an areal capacitance of 470.5 mF cm^−2^, which is much larger than that of the bare carbon (93.8 mF cm^−2^). However, since the insulating nature of Fe_2_O_3_, too thick a coating layer would degrade the rate performance of electrodes. A proper control in ALD coating thickness is thus essential for optimized performance. V_2_O_5_ and Fe_2_O_3_ have been investigated as active anode materials, which are believed to help improve the performance of carbon anodes. Cathode materials (such as NiO, Co_3_O_4_) can also be deposited on carbon support for supercapacitor applications. The carbon support contribute little capacitance in cathode performance. Further discussion will be given in Section 2.2.2, detailing ALD active materials on conductive support.

Transition metal oxides/hydroxides and conducting polymers store charges with surface Faradic reactions, when employed as supercapacitor electrode materials.^57^, ^58^ They generally provide much higher capacitances than those of the carbon‐based materials, although their cycling ability is poor. To improve the stability and capacitance of conducting polymers, Xia et al. deposited ALD RuO_2_ layer on polyaniline (PANI) nanowire surface, where a PANI–RuO_2_ core@shell nanostructure was formed, as shown in Figure 4f–h.^59^ The capacitance, rate capability and cycling stability of PANI were all improved. For example, PANI coated with 100‐ALD‐cycle RuO_2_ showed a specific capacitance of 710 F/g at 5 mV s^−1^, which was much higher than that of PANI alone (564 F g^−1^). More importantly, 88% of its capacitance was maintained after 10 000 cycles at 20 A g^−1^, while it was only 65% being maintained for PANI alone after the same number of cycles. The authors observed that both the capacitance and rate capability of PANI–RuO_2_ could be largely reduced with too thick RuO_2_ coatings. Thus an optimized thickness of ALD RuO_2_ is needed to balance the overall performance including the capacitance, rate and cycling behavior.

#### 2.1.4 ALD for Surface Modification of other Electrochemical Energy Storage Devices

**Figure 5:**
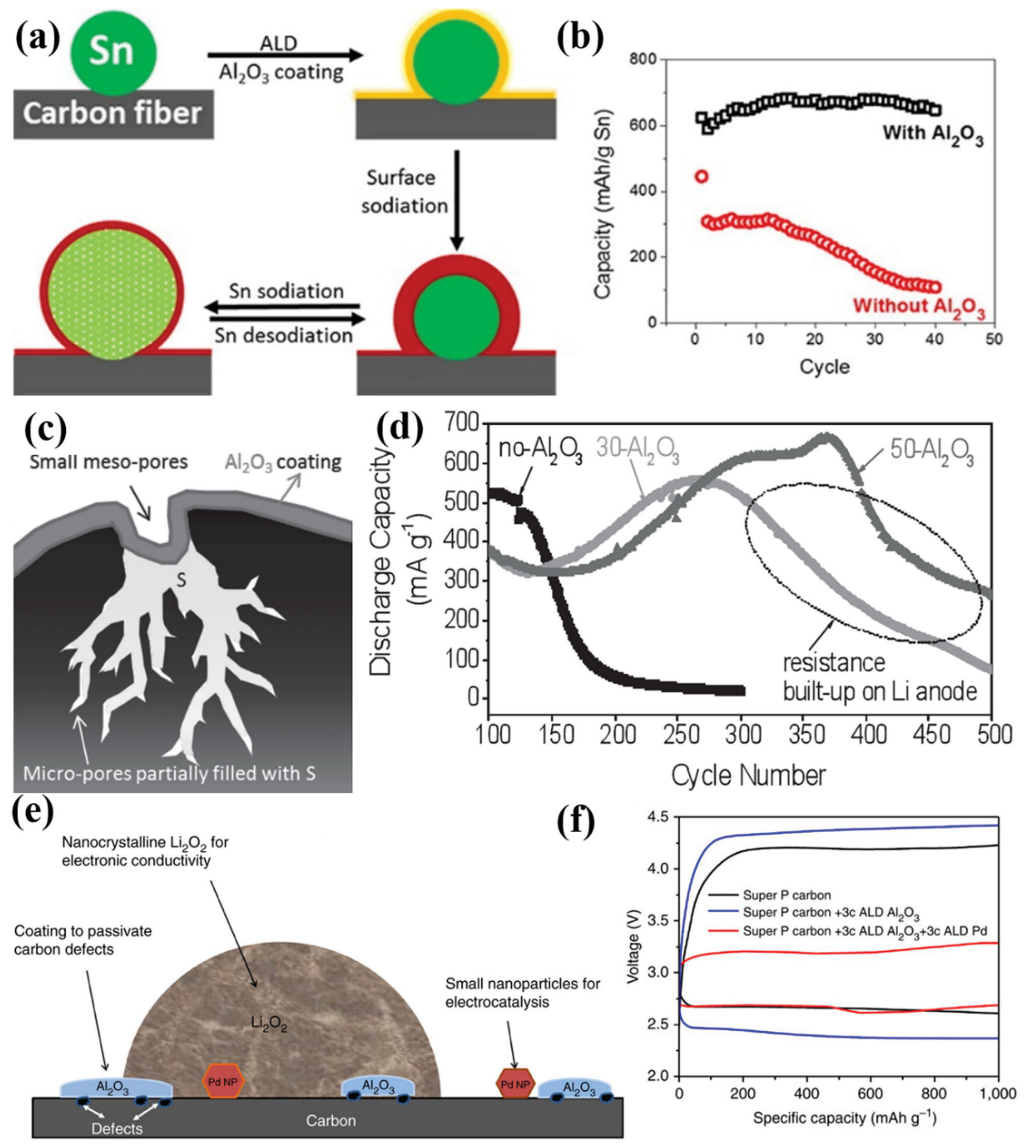
ALD surface modification for electrochemical energy storage systems. a) Schematic processes of ALD Al_2_O_3_ coated Sn nanoparticles on carbon nanofibers for Na ion battery: ALD‐Al_2_O_3_ coating is first uniformly converted to a Na−Al−O layer, then undergo a reversible and stable sodiation/desodiation of the Sn core. b) Cycling ability of the Sn@carbon nanofiber anodes with and without an ALD‐Al_2_O_3_ coating in half‐cell for Na ion battery. c) Schematic of conformal ALD Al_2_O_3_‐coated S/active carbon fibers for Li–S battery. d) Cycling ability of S/active carbon fiber electrodes with different ALD Al_2_O_3_ coating cycles. e) Schematic of ALD Al_2_O_3_ and Pd nanoparticles coated carbon cathode for Li–O_2_ battery. The Al_2_O_3_ coating, the Pd nanoparticles and the nanocrystalline lithium peroxide can contribute to lowering the overpotential. f) Discharge‐charge curves of Li–O_2_ batteries with cathode materials of super P carbon, Al_2_O_3_‐coated super P carbon, and Al_2_O_3_‐coated super P carbon further coated with Pd nanoparticles. a,b) Reproduced with permission.^63^ Copyright 2013, American Chemical Society. c,d) Reproduced with permission.^70^ e,f) Reproduced with permission.^78^ Copyright 2013, Nature Publishing Group.

Na‐ion batteries (SIBs) have redrawn considerable attention in recent years, by considering the abundance and low cost of Na and their promise for large‐scale storage applications.^60^ Although they share similar fundamental principles as LIBs, the Na‐intercalation chemistry and surface modification have not been fully explored. Nevertheless some studies have been made with sodium‐based compounds.^61^ ALD surface coatings on certain SIB electrodes have been shown to effectively improve the device performance. For example, ALD Al_2_O_3_ coating was able to enhance the cycling ability of anode material of Na_2_C_8_H_4_O_4_.^62^ As shown in **Figure** **5**a–b, Han et al. demonstrated that the cycling ability of Sn nanoparticles (as anode for SIB) was significantly improved with an ALD Al_2_O_3_ coating.^63^ With the help of in situ transmission electron microscopy (TEM), the dynamic mechanical protection of ALD Al_2_O_3_ coating was clearly revealed. A unique Na−Al−O layer was formed during the reaction of Al_2_O_3_ and Na ions, which acted not only as a mechanical protection for the Sn inside, but also an ion transport channel for improved Na ions diffusion. The work by Jung et al., who used dynamics calculations, suggested that Na ion diffusivity in Na*_x_*Al_2_O_3_ could be much higher than the Li ion diffusivity in Li*_x_*Al_2_O_3_,^64^ thus the influence of ALD Al_2_O_3_ in SIB was quite different from that in LIB. The high diffusivity of Na ions in Al_2_O_3_ might therefore bring better electrochemical properties for ALD coated electrodes in SIBs.

Due to the high specific energy density in theory and abundance in starting materials, other battery systems, such as Li–S and Li–O_2_ have received considerable attention recently.^18^, ^65^, ^66^ For example, the theoretical capacity of S cathode is 1673 mAh g^−1^, which is much larger than some of those currently known cathodes of metal oxides in LIBs (400 mAh g^−1^).^67^ The theoretical energy density of Li–O_2_ battery is as high as 3505 Wh Kg^−1^, while commercial LIBs can only reach a fraction of this theoretical value.^18^ In Li–S battery, because of the insulating nature of S and the dissolution of polysulfide, the rate and cycling ability are usually rather poor.^68^ Researchers have demonstrated that coupling S or Li_2_S with conductive carbon is an effective pathway to improve the Li–S battery performance, where proper surface coating by ALD contributes to improved electrochemical properties.^69^ ALD Al_2_O_3_ surface coating has been reported by Kim et al., who developed an electrode with sulfur‐infiltrated activated carbon fibers (S–ACFs). As shown in Figure 5c–d,^70^ a significant enhancement in cycling ability was demonstrated. The ALD Al_2_O_3_ coated electrode retained a high specific capacity of 600 mAh g^−1^ after 300 charge–discharge cycles at 0.2 C, while the capacity of the uncoated electrode dropped to almost zero. The much improved cycling ability was attributed to the ALD layer, which effectively confined the polysulfides inside within the barrier thus reduced S dissolution. The reduction in S dissolution has been further confirmed by ex situ SEM and EDS mapping experiment of the S–ACFs electrode and the lithium anode. Other works on ALD surface coating on carbon/S electrodes^71^, ^72^ have also demonstrated much improved cycling ability, in which ALD surface coating is generally utilized as an artificial SEI layer and a barrier layer to prevent the S loss.^73^ For example, ALD Al_2_O_3_ coated on carbon cloth was successfully assembled between S electrode and the separator in the Li–S system.^74^ For the cell made with ALD‐Al_2_O_3_ coated on carbon cloth, the initial discharge capacity was demonstrated 25% higher than the one without ALD coating. 70% of the capacity could be maintained after 40 cycles, which was also much improved compared with the one without ALD coating. SEM and EDX results further confirmed that the coating of ALD‐Al_2_O_3_ on carbon cloth could effectively adsorb and reactivate the dissolved polysulfides from electrolytes.

As another promising type of battery system for energy storage, Li–O_2_ batteries are still in the infancy of their development. There are several scientific and technological challenges in this complex system that need to be addressed in detail,^75^ including for example, the severe passivation and corrosion of anode with low Coulombic efficiency. The electrolyte should be stabilized and have sufficient Li^+^ conductivity and O_2_ solubility. Cathode materials with proper pore structures (e.g., pore size and distribution) and efficient catalysis are needed in order to prevent electrical passivation from discharge products.^65^, ^76^ As an unique surface modification process for electrodes of LIBs, ALD surface coating has been conducted with cathode materials of Li–O_2_ battery.^77^ Utilizing ALD Al_2_O_3_ and Pd, Lu et al. developed a novel cathode material of palladium nanoparticles and thin alumina layer coated on carbon.^78^ As shown in Figure 5e–f, the ALD alumina layer effectively protects the carbon surface and prevents the decomposition of electrolytes, while the nanosized Pd electrocatalyst promotes the formation of nanocrystalline discharge product of Li_2_O_2_, which is beneficial for charge transport. The Pd and Al_2_O_3_ surface‐coated carbon gives rise to a very small charge over‐potential of ≈0.2 V. Wang's group have constructed a hierarchical structure of Li–O_2_ battery cathode, in which three dimensionally ordered mesoporous carbon support was protected by an ALD FeO*_x_* layer, followed by ALD Pd nanoparticles.^79^ In the structure, the carbon support localizes the Li_2_O_2_ deposition, FeO*_x_* protects the inner carbon and catalyses the decomposition of Li_2_O_2_, and Pd nanoparticles serve as an oxygen reduction reaction (ORR) catalyst. With the ALD coating of FeO_x_ and Pd, the cycling ability of the carbon support is improved from 16 to 68 cycles. Liu et al. also developed Pd‐coated, ZnO passivated carbon cathode for Li–O_2_ battery, in which ZnO serves as a protective layer for inner carbon and Pd acts as an effective catalyst for oxygen evolution reaction.^80^ Other efficient catalysts developed by ALD, such as Ru, Pt and Pt_3_Co,^81^ have also been reported for Li–O_2_ battery, with enhanced electrochemical performance.

### 2.2 ALD of Active Materials

Direct deposition of active materials by ALD has led to the development of efficient electrode structures on different substrates. The studies also help understand the fundamental reaction/energy storage mechanisms of the active materials involved. This section will look at ALD active materials developed for improved energy storage, where their merits (such as the tunable thickness, uniformity and conformal surface, and interface with complex substrates) will be discussed. The relationships between the textures of resultant ALD active materials and their performance in electrochemical devices will also be reviewed.

There are several unique and beneficial features of the ultrathin layer materials grown by ALD on 3D complex substrates for electrochemical energy storage. Indeed, several ALD cathode and anode materials for LIBs have been studied, for example including FePO_4_,^82^ Li*_x_*Mn_2_O_4_ and Li*_x_*V_2_O_5_,^83^ LiCoO_2_,^84^ V_2_O_5_,^85^ Co_3_O_4_,^86^ RuO_2_,^87^ SnO_2_,^88^ and TiO_2_.^89^, ^90^ Active materials of metal sulfides,^91^ such as Cu_2_S,^92^ GaS*_x_*,^93^ have also been developed. Similar to metal oxides, ALD metal sulfides utilize metalorganic precursors for metal sources. The difference is however hydrogen sulfide (H_2_S) being used as the typical sulfide source for sulfides, while H_2_O, O_2_ plasma and O_3_ are commonly employed as the oxygen sources for metal oxides. ALD has also been utilized for the deposition of active materials for supercapacitors^94^ and Li–S batteries.^95^ Since the thickness of thus deposited active materials can be well controlled down to atomic layers, the studies help understand the key charge transfer and energy storage mechanisms (such as ion diffusion, absorption as well as redox processes involved) in these active materials.

**Figure 6:**
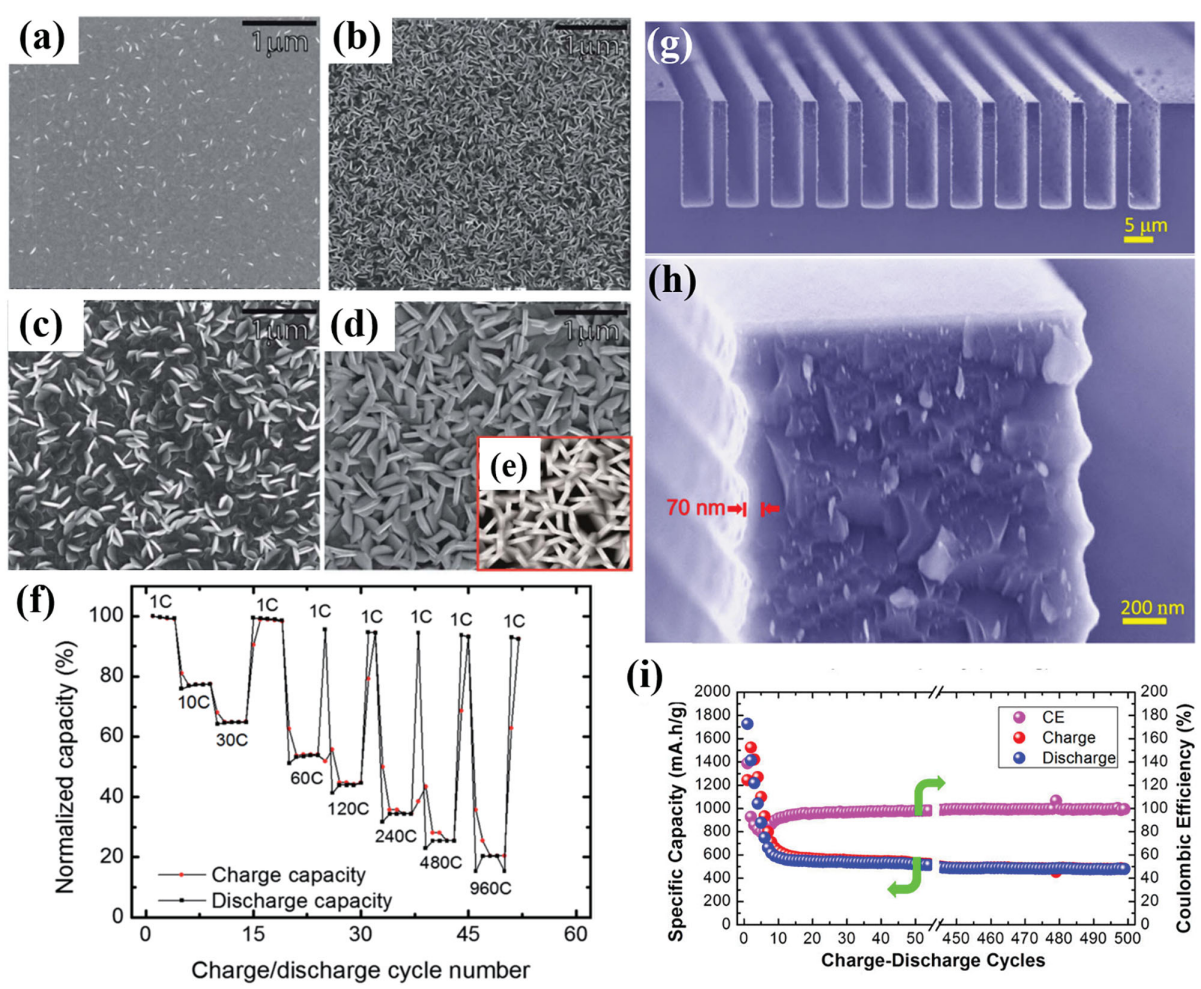
ALD active materials for electrochemical energy storage. a–d) SEM images of ALD V_2_O_5_ with different cycles (500, 1000, 2000, and 5000) on Si substrate. e) Simulation of a surface equivalent to the sample deposited using 5000 cycles. f) Rate performance of 500 cycles of ALD V_2_O_5_ electrode between 1 and 960 C, the capacity retention is normalized to the capacity at 1 C. g–h) SEM images of ALD Li_2_S on Si substrate. i) Cycling ability and Coulombic efficiency of the Li_2_S electrode with 500 cycles. a–f) Reproduced with permission.^96^ Copyright 2014, Royal Society of Chemistry. g–i) Reproduced with permission.^95^ Copyright 2014, American Chemical Society.

Østreng et al. have investigated ALD V_2_O_5_ as the cathode material for LIBs by using VO(thd)_2_ and ozone as the precursors.^96^ As shown in **Figure** **6**a–f, ALD V_2_O_5_ showed a highly textured surface with lots of platelets, which apparently offer large surface area for efficient electrode‐electrolyte contact. In characterization of electrochemical behavior, the electrode with 500‐cycle ALD V_2_O_5_ is discharged with rates up to 960 C, while maintaining 20% of the initial 1 C capacity. It depicted excellent cycling ability, which maintained more than 80% of the initial capacity at more than 1500 cycles at a discharge rate of 120 C. The work has successfully led to an electrode with high cycling stability and high power density, where the confined nanosized V_2_O_5_ and the direct contact with the current collector contribute to the excellent high power and long‐time cycling ability.

In order to develop Li_2_S in a more tunable and controllable manner, which is a promising cathode for Li‐S battery, Meng et al. have successfully synthesised amorphous Li_2_S by ALD using lithium tertbutoxide (LTB, LiOC(CH_3_)_3_) and hydrogen sulfide (H_2_S) as the precursors.^95^ As shown in Figure 6g–i, the resultant Li_2_S is uniformly deposited on the high aspect ratio silicon trench, and the growth rate of Li_2_S is ≈1.1 Å per cycle in the temperature range of 150–300 °C. In the electrochemical characterization, the 700‐ALD‐cycle Li_2_S film was showed with excellent cycling ability for 500 cycles at a high current of 840 mA g^−1^. It also demonstrated a high Coulombic efficiency without the help of the normally used electrolyte additives. Since the conformal nature, ALD is a promising technique for depositing active materials on complex surfaces and 3D substrates, as has been demonstrated with 3D conductive metal substrates and hierarchical carbon supports.

#### 2.2.1 ALD Active Materials on 3D Conductive Metal Nanostructures

**Figure 7:**
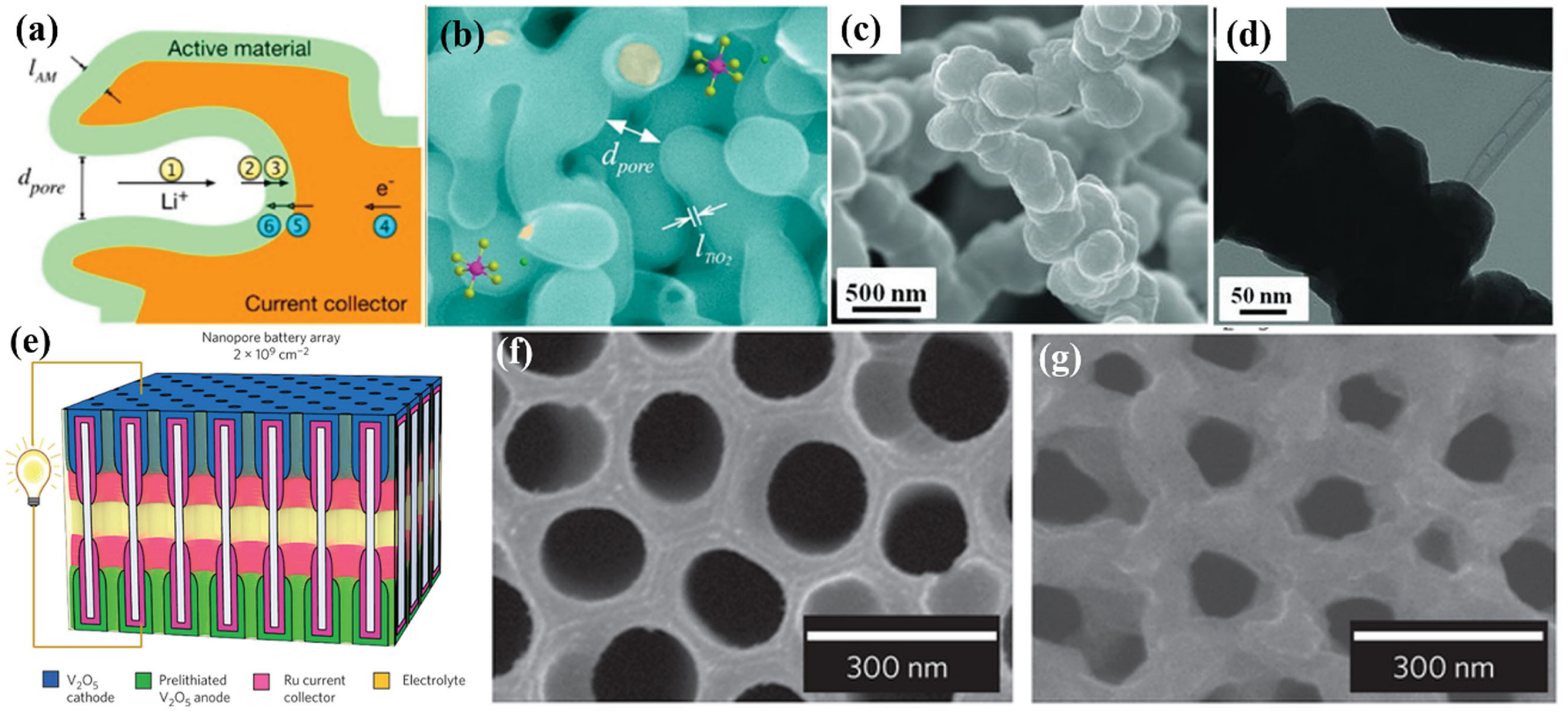
ALD active materials on 3D current collectors for electrochemical energy storage. a) Schematic of transport process of 3D nanoporous gold coated with ALD TiO_2_ electrodes for lithium ion batteries. b) SEM image of the ALD TiO_2_ coated 3D gold. c) SEM and d) TEM images of ALD SnO_2_ coated Ni nanofoam. e) Schematic of parallel nanopore battery array by ALD V_2_O_5_ and Ru on AAO template. f,g) SEM images of AAO coated with ALD Ru (f), and ALD Ru and V_2_O_5_ (g). a,b) Reproduced with permission.^97^ Copyright 2015, American Chemical Society. c,d)Reproduced with permission.^98^ e–g) Reproduced with permission.^100^ Copyright 2014, Nature Publishing Group.

As shown in **Figure** **7**a–b, Ye et al. have succeeded with ALD TiO_2_ deposition on nanoporous gold substrates, where both the pore size and thickness of the active materials can be well controlled for optimised Li^+^ diffusion length in the electrolyte and solid state active materials.^97^ A high power density of 13 KW Kg^−1^ with high energy density of 130 Wh Kg^−1^ is achieved with the sample of 225 nm in pore size and 2 nm TiO_2_ surface coating. The large pore size is essential for Li^+^ diffusion in electrolytes at high rates and the thin layer of TiO_2_ benefits the short Li^+^ diffusion length. Haag et al. successfully fabricated 3D nanostructures of ALD SnO_2_ conformally coated on Ni nanofoam, one example of which is shown in Figure 7c–d.^98^ The 3D Ni nanofoam substrate with high surface area and high conductivity is shown to contribute more sites for fast electrochemical reaction, and the ultrathin layer of ALD SnO_2_ effectively buffers the volume change for long‐time cycling ability. The Ni nanofoam ALD coated with 8 nm SnO_2_ expressed an initial discharge capacity of 546 mAh g^−1^. It could maintain 505 mAh g^−1^ after 100 cycles at a current of 500 mA g^−1^, showing the excellent cycling ability. Other ALD coated 3D metal substrates, such as Ni/TiO_2_, Al/TiO_2_, Ni/V_2_O_5_, have also demonstrated the combined merits from conductive substrate and the conformal coating of thin layers of active materials.^99^

Using anodic aluminum oxide (AAO) for developing nanopores, Liu et al. constructed a novel all‐in‐one battery with parallel nanotubular arrays as electrodes, where the liquid electrolyte is confined within AAO nanopores. As shown in Figure 7e–g,^100^ ALD Ru could be used as nanotube current collectors and ALD V_2_O_5_ served as active material for energy storage. With the elegant device design, the thin layer of V_2_O_5_ was fully exposed to the electrolyte, and the confined connection with nano‐sized Ru current collector drastically facilitated the fast ion and electron transports. In electrochemical characterization, the device was demonstrated with an excellent rate capability with ≈50% of capacity (relative to 1 C) maintained at the 150 C (24 s charge–discharge time), and good cycling ability, with more than 80% of initial capacity being retained after 1 000 cycles at 5 to 25 C.

In general, much improved electrochemical energy storage performance has been demonstrated by ALD active materials deposited on 3D conductive substrates,^101^ where the substrate provides the desired electrical conductivity with high specific surface area, and the ultrathin layer of active materials by ALD enables short ion diffusion length, which benefits the fast electrochemical reaction. The ALD active materials thus deposited on conducting supports are therefore very promising candidates for electrochemical energy devices, for example, as has been demonstrated with 3D all‐solid‐state micro‐batteries.^102^

#### 2.2.2 ALD Active Materials on Carbonaceous Materials

As a typical class of electrode materials for electrochemical energy storage, carbonaceous materials exhibit high electrical conductivity and tunable high surface area, which are essential for high rate performance and high power density. On the other hand, transition metal oxides/hydroxides of high capacity/capacitance are beneficial for high energy density. Therefore a proper construction of hybrid structures combining carbon materials with metal oxides/hydroxides takes the advantages of each constituent components and their synergetic efforts.^57^, ^103^ Considering the conformal and controllable ALD features down to atomic scales, decorating ALD active materials on carbonaceous substrate is of particular interest for much improved device performance.^53^ ALD active materials on conductive carbon support gives rise to shortened ion diffusion length thus benefiting the high power. Together with the well tunable thickness/mass of active materials, they lead to much improved overall electrochemical performance.

**Figure 8:**
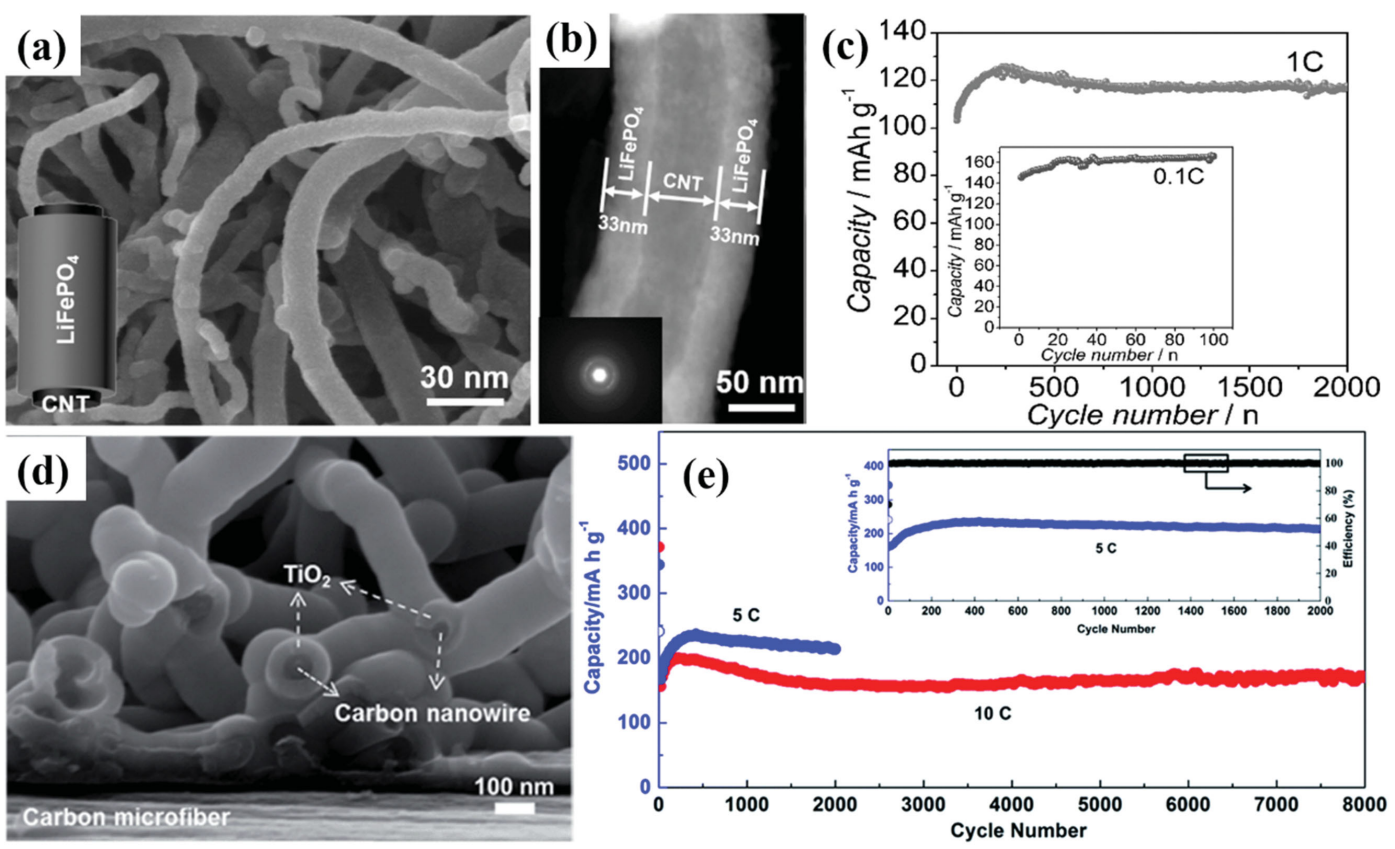
ALD active materials on carbonaceous supports for electrochemical energy storage. a) SEM and b) TEM images of ALD LiFePO_4_ coated CNT. c) Cycling performance of the CNT@LiFePO_4_ cathode for lithium storage. d) SEM image and e) cycling behavior of ALD TiO_2_ coated carbon cloth/carbon nanowire as a LIB anode. a–c) Reproduced with permission.^104^ d,e) Reproduced with permission.^105^ Copyright 2015, Royal Society of Chemistry.

ALD with the quaternary cathode material of LiFePO_4_ on conducting substrates, such as CNTs, has been successfully developed, by using 5 different precursors and carefully tailoring their surface reactions, as shown in **Figure** **8**a–c.^104^ The LiFePO_4_/CNTs electrode thus derived demonstrated a high discharge capacity of ≈150 mAh g^−1^ at 0.1 C. In addition, it also expressed excellent rate property, where a high capacity of 71 mAh g^−1^ was maintained when the current was increased to 60 C. The cell could retrieve the capacity after the current being cycled from 0.1 to 60 C. A key parameter in evaluating the electrode performance is the cycling ability, where the LiFePO_4_/CNTs shows a high capacity retention of 120 mAh g^−1^ after 2000 cycles at 170 mA g^−1^. The excellent rate and cycling ability of LiFePO_4_/CNTs was derived from the unique combination of carbon nanostructure and ALD layer.

Another interesting feature of carbonaceous materials is their mechanical robustness and flexibility. They can be made into 3D supports with flexibility. Using 3D flexible supports made of carbon cloth decorated with carbon nanowire array, Wang et al. further decorated the carbon surface with a thin layer of ALD TiO_2_. It was studied as an anode for LIBs. As shown in Figure 8d–e,^105^ compared with planer substrates, ALD gives rise to a much higher (≈300 times) mass loading of TiO_2_ on the hierarchical 3D carbon cloth/carbon nanofiber substrate, which is essentially desirable for high volume/areal capacity. With the unique 3D design and ALD deposition, the C/TiO_2_ demonstrated a high discharge capacity of 309 mAh g^−1^ at 0.2C with a capacity of 100 mAh g^−1^ being maintained at 20 C. Excellent cycling ability was also achieved for the C/TiO_2_, where a high reversible capacity of 170 mAh g^−1^ could be maintained even after 8000 cycles at 10 C. The excellent electrochemical behavior is believed to originate from the conformal thin layer of ALD TiO_2_, which provides a short ionic diffusion length, and the 3D conductive carbon support of high surface area.

There are several other types of 3D nanostructures of ALD active materials deposited on carbon supports that have been reported,^106^ for example, including the CNT sponge/V_2_O_5_,^107^, ^108^ CNT/Ru,^87^, ^109^ gaphene foam/ZnO,^110^ and porous graphene/NiO.^111^ Because of the well‐controlled ALD thin layer of active materials (desirable for high energy density) and the high electrical conductivity and high surface area of the carbon support (desirable for high power density), they have been shown very promising for achieving high performance energy storage devices.

#### 2.2.3 ALD Parameters and their Functions on Electrochemical Performance

ALD has been utilized in tuning the deposited materials for different crystallinities, textures and mass distribution, which in turn impact the electrochemical properties.^84^ For example, by tuning the deposition temperature, either amorphous or crystallized SnO_2_ nanoparticles can be deposited on graphene nanosheets. Amorphous SnO_2_ showed an apparently better cycling ability since it could better buffer the volume change than the crystallized nanoparticles.^112^

The amount of ALD active materials can well be controlled with different number of deposition cycles, which influence the electrochemical performance. For example, the capacitance of PANI nanowires is largely increased when ALD coated with 100 cycles of RuO_2_, while a surface coating of 1500 ALD cycles coating results in a drastic drop in capacitance (Figure 4h).^59^

Tuning the type of precursors in the 2^nd^ half reaction strongly influences the nature of ALD materials and electrochemical behavior. For example, by changing the plasma precursor from O_3_ to NH_3_, Mattelaer et al. observed a variation between MnO_2_ and MnO, which were employed as cathode and anode for LIB, respectively.^113^

Last but not least, since ALD is based on the surface‐determined chemical reactions, surface properties of substrates greatly influence the morphologies and properties of ALD materials.^114^, ^115^ For example, unmodified carbon surface (e.g., single‐walled carbon nanotubes or graphene) are generally chemically inert to ALD precursor molecules, thus active materials grow at the defect sites.^116^ By proper surface functionalization (such as by conjugating –NO_2_ or –OH groups) on carbon surface, rather uniformly deposited ALD materials are obtained. The substrate surface properties have also shown great influence on electrochemical behavior and other functional behavior such as catalysis and sensing,^115^, ^117^ and therefore they impact on the device performance. To develop a proper continuous layer of ALD TiO_2_ on graphene, ALD Al_2_O_3_ has to be deposited first on graphene as a buffer layer, otherwise only isolated nanoparticles of TiO_2_ are formed with little capacity.^118^

### 2.3 Construction of Advanced Nanostructured Electrodes by ALD

As has been discussed above, ALD has been successfully developed for surface modification of electrodes and deposition of high quality active materials for energy storage, both of which are useful in the rational design and fabrication of electrodes for electrochemical energy storage. In addition, as will be discussed in this section, ALD has been used in development of several new nanostructures, which are otherwise difficult to achieve by conventional processing techniques, including for example 3D types, core@shell types, and hollowed nanostructures. ALD precursors can also be successfully exchanged/transferred into other types of materials for different functionalities, but preserve the desired uniformity and conformity.

#### 2.3.1 ALD for 3D Current Collectors and Structure Support

Since the concept of all‐solid‐state 3D‐integrated batteries was first proposed for miniaturized wireless and portable electronics, ALD has demonstrated its merits in the construction of such 3D structures of high aspect‐ratio for current collectors. For example, ALD TiN is deposited on patterned Si surface as a current collector for Li ion batteries, in which the TiN layer acts as a Li^+^ diffusion barrier.^119^ Other ALD materials, such as TaN and Pt, have also been reported as 3D current collectors.^120^ Besides Si substrate‐based 3D batteries, other micro/nano templates have also been reported where ALD materials are used for current collectors.^121^ As has been discussed previously, ALD Ru has been employed as 3D current collector for nanopore‐based lithium‐ion battery.^100^ Using AAO template and ALD Pt,^122^ Wen et al. constructed Pt@MnO_2_ core‐shell nanotube arrays for supercapacitor electrode, which was showed with high capacitance and rate capability.

**Figure 9:**
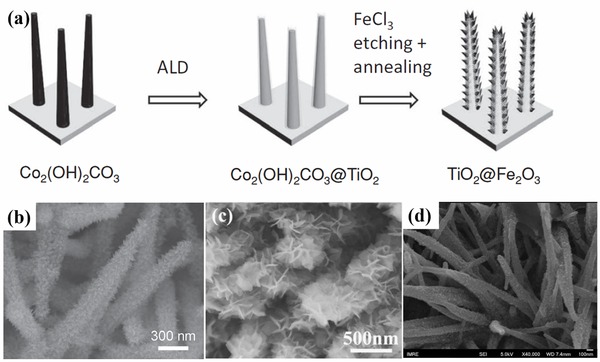
ALD for 3D current collector and structure support. a) Schematic illustration, and b) SEM image of ALD TiO_2_@Fe_2_O_3_ nanostructures. Reproduced with permission.^123^ c) SEM image of ALD TiO_2_@SnO_2_ nanoflakes. Reproduced with permission.^124^ Copyright 2014, Elsevier. d) SEM of ALD TiO_2_@CoS nanoparticles. Reproduced with permission.^125^ Copyright 2015, Royal Society of Chemistry. a,b) Reproduced with permission.^123^ c) . Reproduced with permission.^124^ Copyright 2014, Elsevier. d) Reproduced with permission.^125^ Copyright 2015, Royal Society of Chemistry.

ALD metal oxides have been employed to construct 3D current collectors using nanostructure‐based templates. For example, Luo et al. developed a 3D nanostructure of TiO_2_ nanotube@Fe_2_O_3_ nanoflakes, which was made by ALD TiO_2_ on the surface of Co_2_(OH)_2_CO_3_ followed by a solution growth process.^123^ As shown in **Figure** **9**a–b, ALD TiO_2_ was developed into a uniform tubular structure for fast electron transfer, which would also benefit for the stable deposition of Fe_2_O_3_. The hollowed nanostructure of TiO_2_@Fe_2_O_3_ demonstrated an initial capacity of 840 mAh g^−1^, and a capacity of 530 mAh g^−1^ could be maintained after 200 cycles, which was much enhanced when compared with the electrode without ALD TiO_2_ support. As shown in Figure 9c–d, the ALD TiO_2_ nanotubes could be also combined with other materials, such as SnO_2_ and CoS, which could lead to further enhancement in electrochemical performance.^124^, ^125^ ALD has been successfully employed for fabrication of other 3D active materials,^126^ e.g., SnO_2_@TiO_2_ double‐shell nanotubes, derived from ALD SnO_2_ and TiO_2_ deposited on ZnO nanowire substrate (Figure 2f).^127^

#### 2.3.2 ALD for Construction of Hollow/Porous Nanostructures

Although metal oxides/hydroxides exhibit a theoretical energy density much higher than that of the carbonaceous counterpart, their intrinsically poor electric conductivity is a hindering parameter for application as electrode materials in supercapacitors. In addition, it is more challenging to develop a high surface area and well controlled pore structure for some of these metal oxide/hydroxide electrodes. The volume change and strain generated during the electrochemical reaction can drastically degrade their cycling ability.^128^ Given that properly controlled pore and/or hollowed nanostructures have been demonstrated with enhanced rate capability and cycling stability,^129^ ALD has been explored for construction of some these unique nano‐/micro‐hollow and porous nanostructures.

**Figure 10:**
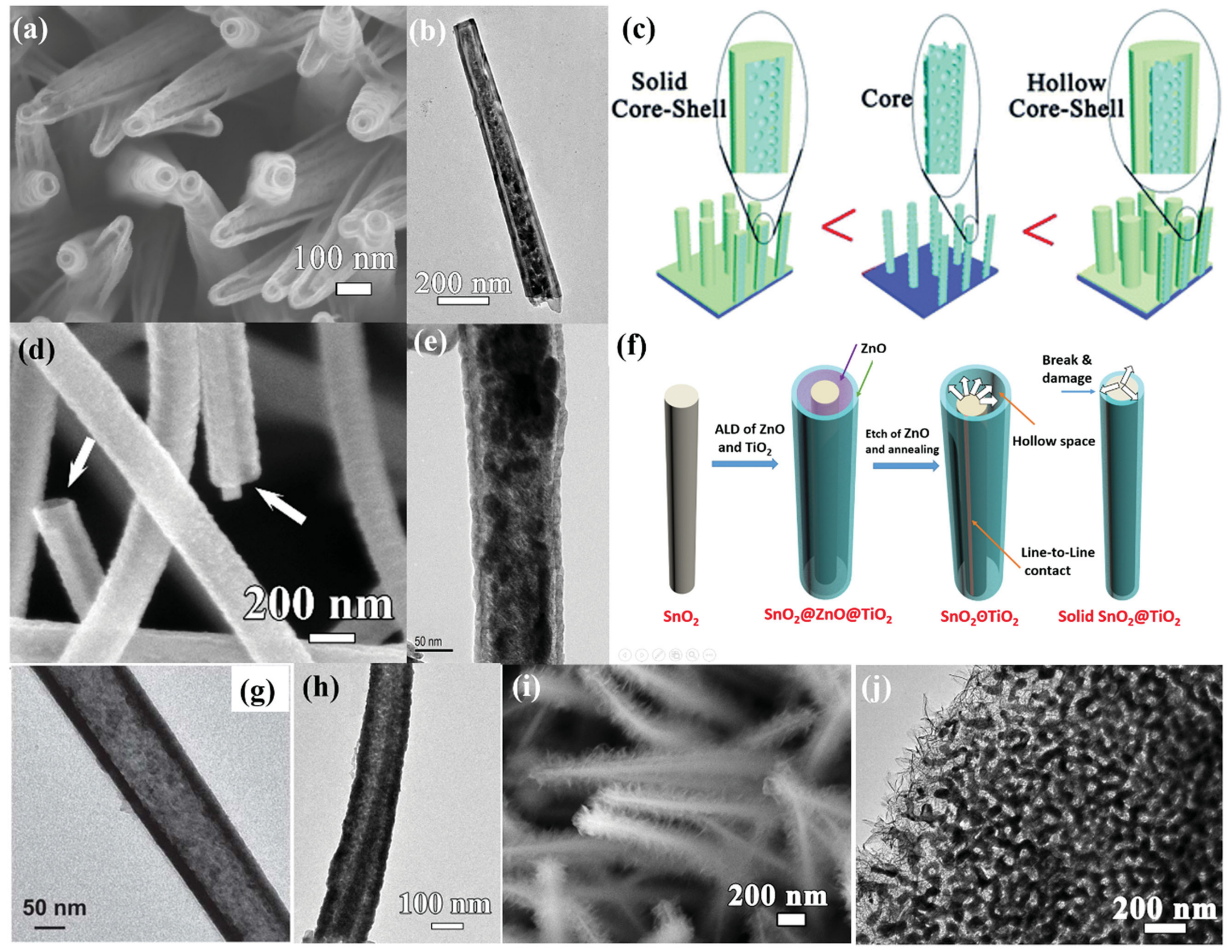
ALD for construction of hollow/porous nanostructures. a) SEM and b) TEM images of CoO⊙TiO_2_ hollowed core‐shell nanowires. c) Schematic illustration of the hollowed core–shell structure in supercapacitor, which is better than the bare core and solid core‐shell structures. d) SEM image of SnO_2_⊙TiO_2_ hollowed wire‐in‐tube nanoarrays. e) TEM image of the hollowed nanostructure after LIB test. f) Schematics of the fabrication process of the SnO_2_⊙TiO_2_ wire‐in‐tube nanostructure, which has free space for volume expansion thus more stable than the solid wires. g) TEM image of a CoO–CoTiO_3_ nanotube. h) TEM image of CoO⊙CoSnO_3_ wire‐in‐tube structure. i) SEM image of CoO@carbon nanoflakes. j) TEM image of Ni*_x_*Co_1–*x*_O@carbon nanoflakes. a–c) Reproduced with permission.^130^ Copyright 2012, Royal Society of Chemistry. d–f) Reproduced with permission.^131^ Copyright 2014, American Chemical Society. g) Reproduced with permission.^134^ Copyright 2013, Royal Society of Chemistry. h) Reproduced with permission.^135^ Copyright 2014, Royal Society of Chemistry. i) Reproduced with permission.^136^ j) Reproduced with permission.^137^ Copyright 2015, IOP Publishing

On the basis of ALD Al_2_O_3_ and TiO_2_, porous and hollowed wire‐in‐tube nanoarrays of CoO⊙TiO_2_ have been reported by Guan et al. As shown in **Figure** **10**a–c,^130^ in such rationally designed nanostructure, porous CoO nanowires are in direct contact with current collectors, where TiO_2_ nanotubes contribute to the increased surface area of the electrode and protect the inner structure. The tunable, small gap between CoO and TiO_2_ are designed as an “ion reservoir'' which facilitates the fast electrochemical reaction at high rates. Compared with CoO nanowires or solid core@shell structure of CoO@TiO_2_, the electrode made of the new nanostructure showed much improved capacitance and rate capability, e.g., two to four times of the capacitance of the solid wires. As shown in Figure 10d–f, based on this new “nanogap” concept, hollowed ALD TiO_2_ nanotubes have been developed for the protection of SnO_2_ nanowires in LIBs.^131^ When employed as an anode material in LIB, alloyed SnO_2_ can generate a huge volume expansion and structure change with poor cycling ability. Although a protective coating layer enhances the cycling ability, the severe structure change can damage the protection layer. The rational design with purposely made hollow nanospace therefore tolerates and buffers the volume change of the inner material thus improving the cycling ability. As has been demonstrated, the SnO_2_⊙TiO_2_ with ≈40 nm gap showed excellent cycling ability, and a capacity of 393.3 mAh g^−1^ is maintained after 1000 cycles. The much improved cycling ability is attributed to the hollow nanospace that buffers the volume change, and the highly stable and uniform ALD TiO_2_ layers stabilize SEI and protect the inner structures.

In addition to the above mentioned examples of new nanostructures developed by ALD, it has been demonstrated useful for creating hollowed nanospace without need for chemical etch of a sacrificed layer, but through solid state diffusion and Kirkendall effect.^132^, ^133^ As shown in Figure 10g, for example, through the solid state diffusion reaction of Co(CO_3_)_0.5_(OH)_0.11_H_2_O nanowire with ALD TiO_2_ layer, Jiang et al. have successfully grown hollowed CoO–CoTiO_3_ nanotube arrays.^134^ Since its unique tubular core‐shell structure and stable ALD CoTiO_3_, the CoO–CoTiO_3_ was illustrated with excellent cycling stability with a capacity of 585 mAh g^−1^ being well maintained after 150 cycles, which is much better than that by CoO. A similar one‐step solid state reaction between Co(CO_3_)_0.5_(OH)_0.11_H_2_O nanowire and ALD SnO_2_ layer results in the formation of CoO nanowires in CoSnO_3_ nanotube structure. As further shown in Figure 10h,^135^ the unique “porous + hollowed” nanostructure of CoO⊙CoSnO_3_ facilitates the electrode/electrolyte contact and provides shortened ion diffusion pathways, thus has demonstrated both better rate and cycling ability than that of CoO alone.

A recent piece of work from Fan's group has investigated several different nanostructures of metal oxide@carbon flakes, one example of which is shown in Figure 10i.^136^ The porous carbon nanoflakes were made by the heat treatment of ALD Al_2_O_3_ and glucose composite layer. The carbon coating provides conductive pathways and high surface area, which are essential for electrochemical electrodes. The carbon nanoflake‐coated CoO nanowires thus constructed showed much better capacitance and cycling ability than those of the bare one, with 98.6% of capacitance being maintained after 5000 cycles. As shown in Figure 10j, the ALD carbon coating nanoflakes can be combined with mesoporous Ni*_x_*Co_1–*x*_O nanosheets, giving rise to much enhanced performance as LIB electrode.^137^

#### 2.3.3 ALD as Sacrificial Layers for Ion Exchange

ALD materials have been employed as sacrificial layers and/or reaction precursors by ion exchange, in deriving other active materials that are otherwise difficult or costly to make. For example, a rather uniform thin film layer of CH_3_NH_3_PbI_3_ perovskite was obtained from ALD PbS with a two‐step ion exchange reaction.^138^ Conformal large‐scale WS_2_ nanosheets are then made by a vapor‐phase ion exchange reaction with ALD WO_3_.^139^ In general, ALD assisted ion exchange involves an in situ reaction, thus the resultant film can well maintain the uniformity of ALD seed layers.

**Figure 11:**
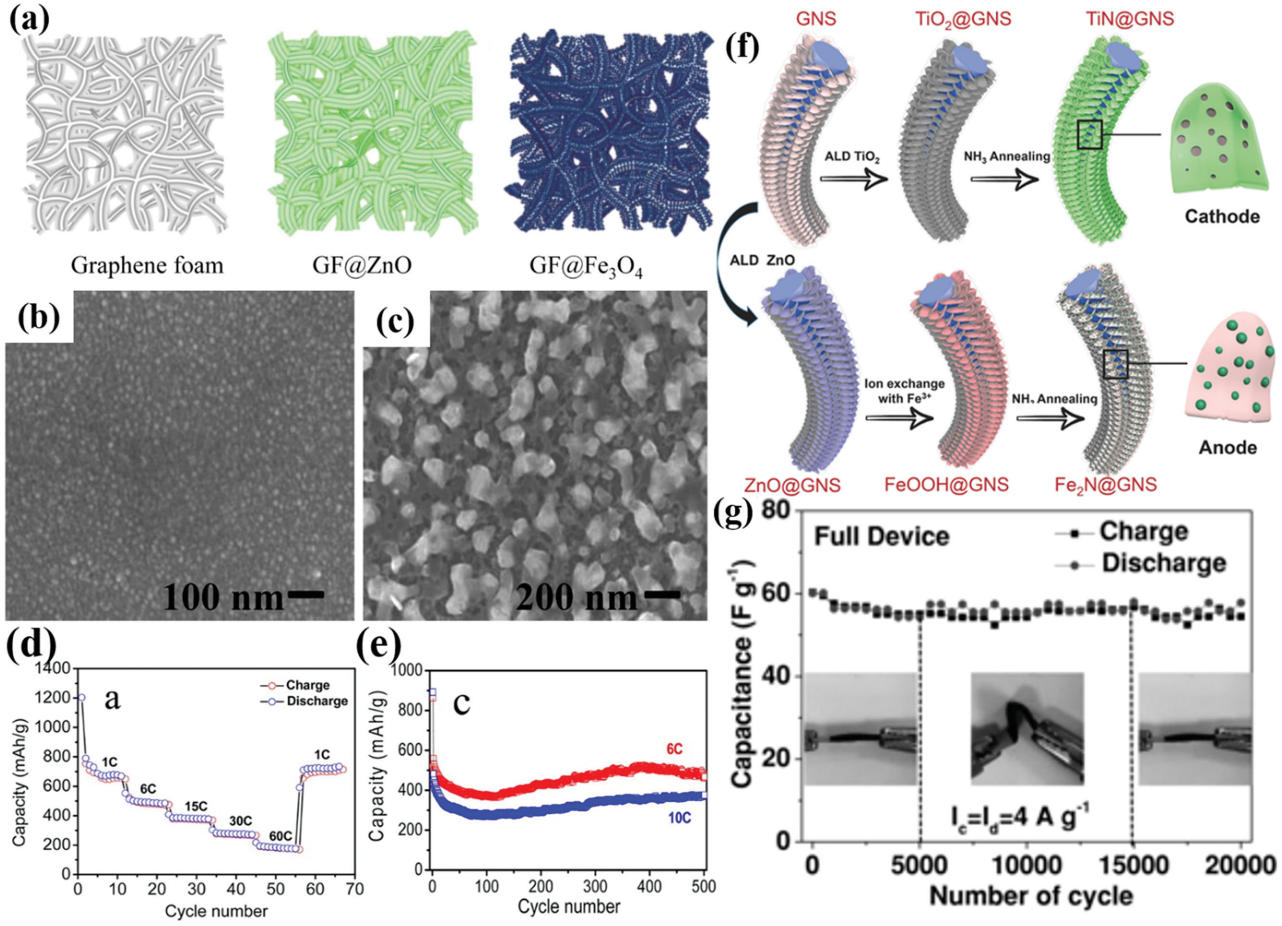
ALD for sacrificial layers in ion exchange. a) Schematic fabrication process of graphene foam (GF)@Fe_3_O_4_ from GF@ALD–ZnO. SEM images of b) GF@ZnO and c) GF@Fe_3_O_4_. d) Rate and e) cycling properties of GF@Fe_3_O_4_. f) Schematics of the fabrication processes of TiN cathode and Fe_2_N anode materials from ALD TiO_2_ and ALD ZnO, respectively. g) Cycling performance of metal nitride full device with different bending situations. a–e) Reproduced with permission.^140^ Copyright 2013, American Chemical Society. f,g) Reproduced with permission.^141^

Through a solution ion exchange reaction with ALD ZnO, Luo et al. have successfully constructed Fe_3_O_4_ nanoparticles on 3D graphite foam, as shown in **Figure** **11**a–e.^140^ The Fe_3_O_4_ nanoparticles were uniformly deposited on the surface of carbon substrate, which facilitated ion transport. In characterization for device performance in LIB, the electrode expressed a high capacity of 785 mAh g^−1^ at 1 C rate, and the capacity was well maintained after 500 cycles at 10 C rate. The same ion exchange concept has been utilized for electrodes of supercapacitors. For example, Zhu et al. developed metal nitride solid‐state asymmetric supercapacitors using carbon cloth/graphene nanosheets substrate, one example of which is shown in Figure 11f–g.^141^ Through the ion exchange reaction with ALD TiO_2_ and ZnO layer, thin TiN nanolayers (cathode) and Fe_2_N nanoparticles (anode) were uniformly assembled on graphene nanosheets, which provided a high surface area for fast electrochemical reaction. Using PVA/LiCl electrolyte, the full cell was demonstrated to provide a high energy density of 15.4 Wh kg^−1^ and a high power density of 6.4 kW kg^−1^, together with excellent rate capability and cycling stability.

## 3 Conclusions and Outlook

This review has focused on the recent advances of ALD for new and improved electrode materials in electrochemical energy storage devices. High performance electrochemical energy storage has been extensively developed in recent years, with the typical key performance parameters being the high energy density, high power density and long cycling life stability. Novel electrode materials are crucial for development of the next generation high performance electrochemical energy storage devices with these superior parameters. ALD has been advancing rapidly over the past few years as a powerful nanotechnology for design and fabrication of advanced electrode materials with some of the most desirable features, which cannot be realized by other processing techniques. In this connection, this review has summarized three main aspects: (i) ALD provides a unique platform for surface modification of electrode materials, leading to much enhanced rate capability and cycling stability; (ii) Active materials grown by ALD on different substrates giving rise to some of the most optimized combination of electrochemical properties, where some of the reaction mechanisms and underlying principles have been visited; (iii) ALD has been successfully developed in the rational design and construction of novel nanostructures, which are otherwise impossible/difficult to achieve by other techniques, for electrochemical energy storage.

For the past several years, although considerable progress has been made with ALD for advanced electrode materials, it remains much room for further improvement and key understandings. One promising direction that has been undertaken and will continue is to develop novel ALD materials for surface modification on electrodes, which will bring new surface and interface chemistry for better protection, ion transport and electrochemical reactions. As has been mentioned in this review, since the side reaction and instability of LIB electrodes, ALD Al_2_O_3_ has been often employed for surface coating on these electrodes, while some other works suggest that LiAlO_2_ and CeO_2_ can lead to even further improved performance over Al_2_O_3_. There is still considerable amount of further research needed, in order to properly manipulate the ALD coating layer optimized for different electrochemical energy storage devices, such as ALD oxides, fluorides, phosphates, and Li‐containing coating materials.

Another interesting direction would be to develop new novel active materials by ALD for much improved electrochemistry, which can not only help make better fundamental understanding, but also achieve optimized device performance. It is commonly known that the performance by most of the known active materials is much below their theoretical expectations. Therefore there is still a way to go towards the most desirable structure at varying scales and dimensions. Since ALD gives much better control in materials growth, it will play an important role in the drive towards this goal. Since ALD active materials can be very controlled, they will pave the way towards further understandings on the electrochemical reaction mechanisms and phenomenon in some of these materials, for example by in situ studies.

A further interesting, and equally important future development is the advance of ALD, either by itself or by combination with other processes, in development of completely new nanostructures. Some of the known examples have been mentioned in this review. It will be definitely continue for development of novel materials with new structure for the next generation electrochemical energy storage devices.

Last but not least, with the unique and new electrode structures developed by ALD, it would be of interest to revisit even some of the “old” battery systems, such as aqueous nickel–zinc battery, which may well bring up much higher power density and improved cycling ability.

While ALD has been widely studied for design and construction of advanced electrode materials, the present and future trends are to establish various new phenomena and underlying principles, not only for energy storage, but also for energy generation and environmental devices, such as PVs and catalysts. With the structure, performance and underlying principles be established for materials developed by ALD, it would be of interest to develop new ALD systems for large mass production and at low cost for the expected wide range of applications.^142^

With the steady establishment of ALD, another deposition technique using organic precursors is the molecular layer deposition (MLD), which has shown some potential for electrode surface modification.^143^ The technique, together with use of certain organic compounds, can bring in new electrochemical, electrical, optical, magnetic and catalytical properties. It would also be useful for some of the functional organic−inorganic hybrid materials that can be utilized for electrochemical energy storage.
